# Spatio-temporal Responses of *Arabidopsis* Leaves in Photosynthetic Performance and Metabolite Contents to *Burkholderia phytofirmans* PsJN

**DOI:** 10.3389/fpls.2016.00403

**Published:** 2016-03-30

**Authors:** Fan Su, Françoise Gilard, Florence Guérard, Sylvie Citerne, Christophe Clément, Nathalie Vaillant-Gaveau, Sandrine Dhondt-Cordelier

**Affiliations:** ^1^Unité de Recherche Vignes et Vins de Champagne – EA 4707, SFR Condorcet FR CNRS 3417, UFR Sciences Exactes et Naturelles, Université de Reims Champagne-ArdenneReims, France; ^2^UMR CNRS-INRA 9213, Saclay Plant Sciences, Institute of Plant Sciences Paris-Saclay IPS2 (Bâtiment 630), Université Paris-SudOrsay, France; ^3^Institut Jean-Pierre Bourgin, UMR 1318 INRA-AgroParisTech, ERL 3559 CNRS, INRA Versailles-GrignonVersailles, France

**Keywords:** *Burkholderia phytofirmans* strain PsJN, hormone, metabolite profiling, PGPR, photosynthesis, primary metabolite

## Abstract

A valuable strategy to improve crop yield consists in the use of plant growth-promoting rhizobacteria (PGPRs). However, the influence of PGPR colonization on plant physiology is largely unknown. PGPR *Burkholderia phytofirmans* strain PsJN (*Bp* PsJN) colonized only *Arabidopsis thaliana* roots after seed or soil inoculation. Foliar bacteria were detected only after leaf infiltration. Since, different bacterial times of presence and/or locations in host plant could lead to different plant physiological responses, photosynthesis, and metabolite profiles in *A. thaliana* leaves were thus investigated following leaf, root, or seed inoculation with *Bp* PsJN. Only *Bp* PsJN leaf colonization transiently decreased cyclic electron transport and effective quantum yield of photosystem I (PSI), and prevented a decrease in net photosynthesis and stomatal opening compared to the corresponding control. Metabolomic analysis revealed that soluble sugars, amino acids or their derivatives accumulated differently in all *Bp* PsJN-inoculated plants. Octanoic acid accumulated only in case of inoculated plants. Modifications in vitamin, organic acid such as tricarboxylic acid intermediates, and hormone amounts were dependent on bacterial time of presence and location. Additionally, a larger array of amino acids and hormones (auxin, cytokinin, abscisic acid) were modified by seed inoculation with *Bp* PsJN. Our work thereby provides evidence that relative short-term inoculation with *Bp* PsJN altered physiological status of *A. thaliana* leaves, whereas long-term bacterization triggered modifications on a larger set of metabolites. Our data highlighted the changes displayed during this plant–microbe interaction to trigger physiological and metabolic responses that could explain the increase in plant growth or stress tolerance conferred by the presence of *Bp* PsJN.

## Introduction

Plants are commonly colonized by microorganisms. Non-pathogenic microorganisms, such as PGPR, promote plant growth and improve plant resistance against biotic or abiotic stress (Agrawal et al., 2015; Yadav et al., 2015). Plant growth promotion and improvement of nutrient element acquisition induced by PGPRs could be the direct results of enhanced photosynthesis due to increased chlorophyll a and/or b contents and/or a better PS II activity (Esitken et al., 2003; Zhang et al., 2008; Cohen et al., 2015). Root colonization by the PGPR *Pseudomonas fluorescens* strain Aur6 promoted Fv/Fm and PSII electron transport rate (ETRII) in *Pinus halepensis* Mill. (Rincón et al., 2008). *Bacillus subtilis* GB03 increased *Arabidopsis thaliana* chlorophyll contents, chloroplast number, and photosynthetic capacity through production of volatile organic compounds (Zhang et al., 2008). Foliar spray of *Bacillus* OSU 142 on apricot (*Prunus armeniaca* L. cv. *Hacihaliloglu*) increased nutrient element composition (N, P, K, Ca, and Mg) and also chlorophyll contents (Esitken et al., 2003). Moreover, siderophores produced by bacteria chelate iron and make it available to the plant (Harrington et al., 2015). In plants, iron is involved in the synthesis of chlorophyll, thylakoids, and chloroplasts (Miller et al., 1995). Inoculation of the siderophore-producer *Pseudomonas* strain RRLJ 008 on seeds thus improved chlorophyll contents and crop mass in eggplant, cabbage, French bean, kohlrabi, and tomato (Boruah and Kumar, 2002). Since, carbohydrates produced *via* photosynthesis not only supply carbon and energy but also act as signal molecules involved in plant growth, development, and responses to stresses (Rolland et al., 2002), bacterial endophytes could exert their beneficial effects on plant growth and health by modifying plant photosynthetic activities and thus carbohydrate partitioning (Bolton, 2009; Van Hulten et al., 2010).

Optimal growth and development of plants were also regulated by plant hormones, including gibberellins, auxins, cytokinins, abscisic acid (ABA), and ethylene. Gibberellins, auxins, and cytokinins promote cell division and plant growth but delay leaf senescence (Davies, 2010). Inoculation of plants with PGPRs capable to produce such hormones could thus increase plant biomass and yield (Cohen et al., 2009). In contrast, ABA and ethylene are generally described as plant growth inhibitors and low levels of these hormones are necessary for normal development of plants (Davies and Jones, 1991; Wen, 2015). Some PGPRs thus promote plant growth by reducing ethylene contents through the activity of their 1-aminocyclopropane-1-carboxylic acid deaminase, which metabolizes the precursor of ethylene (Zhou et al., 2013; Wen, 2015). However, growth of maize plants is even thought to be promoted by inoculation of the ABA producer *Azospirillum lipoferum* (Cohen et al., 2009). Moreover, endophytic PGPRs are capable to catabolize some plant compounds. For example, the nitrogen-fixing endophyte *Klebsiella pneumoniae* 342 possessed a large set of genes devoted to transport or to metabolize plant cellulosic and aromatic compounds. Furthermore, accumulation of callose, lignin, or phenolic compounds induced by PGPRs resulted in reinforced plant cell walls (Benhamou et al., 1996; Weston et al., 2012).

The endophytic PGPR *Bp* PsJN colonized and promoted the growth of a wide range of plants (Pillay and Nowak, 1997; Ait Barka et al., 2006; Naveed et al., 2014), including *A. thaliana* (Poupin et al., 2013; Su et al., 2015). This bacterium has been detected in roots and stems of different cultures but also in leaves of grapevine (Compant et al., 2008; Lo Piccolo et al., 2010), maize (Naveed et al., 2014), switchgrass (Kim et al., 2012), and *A. thaliana* (Poupin et al., 2013). *A. thaliana* seed inoculation with *Bp* PsJN induced beneficial effects through producing and regulating indole-3-acetic acid (IAA) levels (Zuniga et al., 2013) and increasing chlorophyll content (Poupin et al., 2013). *Bp* PsJN-inoculated *A. thaliana* plants also displayed an accelerated plant development (Poupin et al., 2013). Considering that bacterial presence may influence plant responses, we hypothesized that presence of PGPRs either in leaves (local place) or roots (distal place) at relatively long-time (weeks) or short-time (days) could lead to different physiological responses in plant leaves. In order to decipher the influence of endophyte activities on plant physiology, biochemical and physiological changes on *A. thaliana* leaves induced by *Bp* PsJN inoculation were analyzed. Three modes of bacterial inoculation, leading to different bacterial locations and times of presence, were thus performed. PSI and PSII activities, gas exchanges and analyses of primary and secondary metabolites were also performed. All these measurements provided an overall snapshot of the physiological responses in *A. thaliana* leaves to the presence of *Bp* PsJN in different locations (local or distal) and at different times (few days to several weeks).

## Materials and Methods

### Plant Material and Growth Conditions

All experiments were performed on 6-weeks-old wild type *A. thaliana* ecotype Col-0. Seeds were sown on soil. Plants were grown in a controlled environment chamber at 20°C, with 60% of relative humidity and a 12 h photoperiod (photosynthetically active radiation, PAR = 120 μmol m^-2^ s^-1^).

### Bacterial Inoculum and Inoculations *In Planta*

Bacterial inocula were performed as previously described by Su et al. (2015). Briefly, *Bp* PsJN tagged with green fluorescent protein (Sessitsch et al., 2005) was grown for 24 h at 28°C at 180 rpm in King’s B liquid medium supplemented with kanamycin and cycloheximide (50 μg mL^-1^). Bacteria were collected after centrifugation at 4,500 *g* for 10 min and suspended in phosphate-buffered saline (PBS, 10 mM, pH 7.2) for seed inoculation or soil irrigation, or in 10 mM MgCl_2_ for leaf infiltration. The concentration of bacterial inocula was adjusted to 600 nm (Pillay and Nowak, 1997) to obtain around 10^4^ to 10^5^ colony forming units per mL (cfu mL^-1^) in leaves inoculated by seed immersion (Bp ST), soil drenching (Bp RT) or leaf infiltration (Bp LT). Control inoculations were performed with PBS in the treatments of seeds (ST) and soil (RT) and with a solution of 10 mM MgCl_2_ in leaf treatment (LT). ST, RT, and LT plants were watered twice a week, with exception of the time during soil treatment for RT plants.

Photosynthesis analysis and sampling for metabolite analyses were carried out on 42-days-old plants treated on seeds (Bp ST or Mock ST). For plants treated by soil drenching (Bp RT or Mock RT) or by leaf infiltration (Bp LT or Mock LT), measurements were performed before (0 day) and 1 (corresponding to 42-days-old plants), 2, 3, 5, and 10 days after bacterial inoculation. For all experiments, three independent biological replicates were performed, each with three plants per treatment.

### Photosynthesis Analysis

#### Fluorescence and P700+ Signal

Photosystems I and II activities in leaf were synchronously measured using a dual-wavelength pulse-amplitude-modulated fluorescence monitoring system (Dual-PAM-100, Walz, Effeltrich, Germany). The Dual-PAM-100 system can detect P700+ absorbance changes and chlorophyll *a* fluorescence at the same time by saturation pulse method after 30 min dark adaptation (Pfündel et al., 2008). Parameters were automatically calculated by the Dual-PAM-100 software during the measurement (Pfündel et al., 2008). Y(CEF) is calculated as difference between the quantum yield of PSI and PSII, Y(CEF) = ΦPSI-ΦPSII (Huang et al., 2010).

#### Gas Exchanges

Net photosynthesis, Ci, gs, and E of leaves were measured simultaneously by an open gas exchange system (LI-6400-XT, LI-COR, Lincoln, NE, USA) fitted with a leaf chamber fluorometer (6400-40, LI-COR, Lincoln, NE, USA), using equations developed by von Caemmerer and Farquhar (1981). CO_2_ concentration was maintained at a constant level of 380 μmol mol^-1^ by using a CO_2_ injector (LI-6400-01, LI-COR, Lincoln, NE, USA) with a high-pressure liquid CO_2_ cartridge source. Air temperature and relative humidity were maintained at 20°C and 40%, respectively. Photosynthetic active radiation was fixed at 200 μmol m^-2^ s^-1^.

### Metabolite Analysis

#### Gas Chromatography–Mass Spectrometry

The relative levels of metabolites (amino acids, sugars, sugar alcohols, and organic acids) in the leaves were determined in an untargeted manner. Extraction and gas chromatography–mass spectrometry (GC–MS) analyses were performed as described by Tcherkez et al. (2009). Leaf samples (20 mg of powder from freeze-dried material) were ground in a mortar in liquid nitrogen and then in 2 mL of 80% methanol in which ribitol (100 μmol L^-1^) was added as an internal standard. After centrifugation, aliquots of each extract (0.2 mL) were spin-dried under vacuum. The extracts were dissolved with methoxyamine (in pyridine) and *N*-methyl-*N* (trimethyl-silyl) trifluoroacetamide (MSTFA). The derivatization mixture was then incubated for 2 h at room temperature. Before loading into the GC autosampler, a mix of a series of eight alkanes (chain lengths: C_10_ to C_36_) was included. Analyses were performed by injecting 1 μL in splitless mode at 230°C (injector temperature). Gas chromatography coupled to time-of-fight mass spectrometry was performed on a LECO Pegasus III with an Agilent (Massy, France) 6890N GC system and an Agilent 7683 automatic liquid sampler. The column was an RTX-5 w/integra-Guard (30 m × 0.25 mm internal diameter + 10 m integrated guard column; Restek, Evry, France). The chromatographic separation was performed in helium as a gas-carrier at 1 mL min^-1^ in the constant flow mode and using a temperature ramp ranging from 80 to 330°C between 2 and 18 min, followed by 6 min at 330°C. Electron ionization at 70 eV was used and the MS acquisition rate was 20 spectra s^-1^ over the m/z range 80–500 as described by Weckwerth et al. (2004). Peak identity was established by comparison of the fragmentation pattern with MS available databases (Fanucchi et al., 2012), using a match cut-off criterion of 700/1000 and by retention time using the alkane series as retention standards. The integration of peaks was performed using the LECO Pegasus (Garges-lès-Gonesse, France) software. Because automated peak integration was occasionally erroneous, integration was verified manually for each compound in all analyses. As a quality control filter, samples were checked for the presence of a strong ribitol peak with a peak area of at least 35000 and a deviation from the median internal standard peak area (for that GC/MS batch sequence) of less than 15% of the median value. Metabolite contents are expressed in arbitrary units (semi-quantitative determination). Peak areas determined using the LECO Pegasus software have been normalized to fresh weight and ribitol area (internal standard).

#### Liquid Chromatography–Mass Spectrometry

In addition to GC–MS analyses, liquid chromatography–mass spectrometry (LC–MS) analyses were performed. Each dry extract was dissolved in 140 μL of acetonitrile/water (50/50; v/v), filtered, and analyzed using a Waters Acquity UPLC-ESI-MS. Since same samples were analyzed by LC–MS and UPLC-ESI-MS/MS, ABA was used as internal standard for LC–MS analyses. The compounds were separated on a reverse-phase column (Kinetex XB-C18, 100 mm × 2.1 mm × 1.7 μm particle size; Phenomenex, France) using a flow rate of 0.4 mL min^-1^ and a binary gradient: (A) formic acid 0.1% in water (v/v) and (B) acetonitrile with 0.1% formic acid. We used the following binary gradient (t, % A): (0 min, 99%), (2 min, 55%), (3 min, 55%), (4 min, 0%), (6 min, 0%), (7 min, 99%), (9 min, 99%), and the column temperature was 30°C. Mass spectrometry was conducted in electrospray, in positive mode for the chlorogenic acid and in negative ion mode for the other molecules. Relevant instrumental parameters were set as follows: capillary 1.5 kV in positive mode and 3.7 kV in negative mode, source block and desolvation gas temperatures 195°C. Nitrogen was used to assist the cone and desolvation (four bars and 8.5 L min^-1^, respectively). Standard mixtures at different concentrations were injected several times throughout the analysis. The reproducibility of their peak areas was checked by superposition of chromatograms and plotting calibration curves.

### Ultra-performance Liquid Chromatography Coupled with Electrospray Ionization/Quadrupole-Time-of-Flight Mass Spectrometry

For these analyses, samples were collected at 0, 3, and 10 dpi in order to obtain a general view of hormonal changes. Salicylic acid, jasmonic acid, ABA, and IAA were determined according to Li-Marchetti et al. (2015). Briefly, leaf samples (10 mg of powder from freeze-dried material) were extracted with 0.8 mL of acetone/water/acetic acid (80/19/1 v:v:v). Those four hormone stable labeled isotopes used as internal standards were prepared as described in Le Roux et al. (2014). Two ng of each standard were added to the sample. The extract was vigorously shaken for 1 min, sonicated for 1 min at 25 Hz, shaken for 10 min at 4°C in a Thermomixer (Eppendorf) and then centrifuged. The supernatants were collected and the pellets were re-extracted twice with 0.4 mL of the same extraction solution, then vigorously shaken (1 min) and sonicated (1 min; 25 Hz). After the centrifugations, the three supernatants were pooled and dried (final volume 1.6 mL). Each dry extract was dissolved in 140 μL of acetonitrile/water (50/50; v/v), filtered, and analyzed using a Waters AcquityUPLC-ESI-MS/MS. The compounds were separated on a reverse-phase column (Uptisphere C18 UP3HDO, 100 mm × 2.1 mm × 3 μm particle size; Interchim, France) using a flow rate of 0.4 mL min^-1^ and a binary gradient: (A) acetic acid 0.1% in water (v/v) and (B) acetonitrile with 0.1 % acetic acid. The gradient was applied as follow (t, % A): (0 min, 98%), (3 min, 70%), (7.5 min, 50%), (8.5 min, 5%), (9.6 min, 0%), (13.2 min, 98%), (15.7 min, 98%), and the column temperature was 40°C. Mass spectrometry was conducted in electrospray and Multiple Reaction Monitoring scanning mode (MRM mode), in positive mode for IAA and in negative ion mode the other hormones. Relevant instrumental parameters were set as follows: capillary 1.5 kV (negative mode), source block and desolvation gas temperatures 130 and 500°C, respectively. Nitrogen was used to assist the cone and desolvation (150 and 800 L h^-1^, respectively), argon was used as the collision gas at a flow of 0.18 mL min^-1^. The parameters used for MRM quantification of the different hormones are described in Le Roux et al. (2014). Samples were reconstituted in 140 μL of 50/50 acetonitrile/H_2_O (v/v) per mL of injected volume. The limit of detection and limit of quantification were extrapolated for each hormone from calibration curves and samples using quantify module of MassLynx software, version 4.1.

Since LC–MS and UPLC-ESI-MS/MS were performed on a small array of samples, few batch effects could be observed. However, samples were randomly injected throughout the analysis in order to avoid batch effects. No outlier has been detected.

### Statistical Analyses

All experiments were repeated independently three times. For metabolite analysis, student’s *t*-tests were performed with normalized data (mean-center) using MeV 4.1 open source software (Saeed et al., 2003) so as to compare leaf infiltration (LT), soil drenching (RT), or ST with their respective control. Metabolites and photosynthesis parameters considered to vary significantly between treatments were those with *P* < 0.05 using Student’s *t*-tests.

## Results

### Colonization of *A. thaliana* Leaf by *Bp* PsJN

We previously showed that *Bp* PsJN bacterization of seeds or by soil irrigation led to root endophytic colonization of *A. thaliana* plants (0.4 × 10^4^ or 1.7 × 10^4^ cfu g^-1^ FW, respectively), and that no bacteria were detected inside and on surface of leaves (Su et al., 2015). Here, *Bp* PsJN leaf infiltration was also employed to test whether physiological modifications induced by the presence of bacteria could be dependent on its location. No symptom was visible in leaves after infiltration (Supplementary Figure S2). Whereas 5.5 × 10^4^ cfu g^-1^ FW was found immediately after leaf infiltration (endophytic and epiphytic bacteria, Supplementary Figure S1), only 0.9 × 10^4^ cfu g^-1^ FW bacteria were detected after leaf surface sterilization (endophytic bacteria). Maximum of endophytic population (around 10^5^ cfu g^-1^ FW) was present 4 days post (bacterial) inoculation (dpi) and was maintained until 10 dpi. Taking together, we thus considered that *A. thaliana* leaf infiltration with *Bp* PsJN could lead to a response at local and short-term levels, whereas root or seed inoculation with *Bp* PsJN results in a response at distal and short- or long-term levels, respectively.

### Modifications in Photosynthesis Are Only Induced by the Presence of *Bp* PsJN in Leaves

#### Photochemistry of PSI and PSII

**FIGURE 1:**
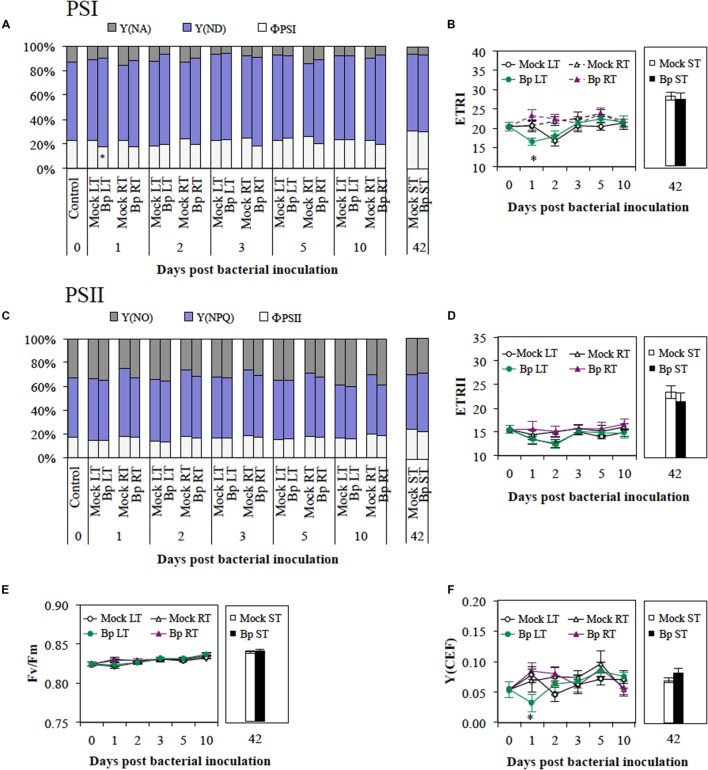
**Photosystem I and photosystem II photochemistry in mature leaves after leaf infiltration (LT), soil drenching (RT), or seed inoculation (ST) with *Burkholderia phytofirmans* strain PsJN.** PSI acceptor side limitation, Y(NA), PSI donor side limitation, Y(ND), efficient quantum yield of PSI, ΦPSI **(A)**, PSI electron transport rate, ETRI **(B)**, quantum yield of non-regulated energy dissipation in PSII, Y(NO) and regulated energy dissipation in PSII, Y(NPQ), efficient quantum yield of PSII, ΦPSII **(C)**, PSII electron transport rate, ETRII **(D)**, maximum photochemical efficiency of PSII, Fv/Fm **(E)**, and cyclic electron flow around PSI, Y(CEF; **F)** were analyzed. Data (mean ± SE) are averages of three experimental replicates, each with three plants per treatment (*n* = 9). *, #, or aaa,indicate significant differences in Bp-LT, -RT, or -ST conditions compared with their respective control, respectively (Student’s *t*-test; *P* < 0.05).

Excitation energy transferred to the PSI centers will result in photochemical charge separation with quantum yield ΦPSI or in non-photochemically conversion to heat (Nelson and Yocum, 2006). The quantum yield of non-photochemical energy dissipation can be due to limitation of the acceptor-site Y(NA) or the donor-site Y(ND). At 1 dpi, *A. thaliana* leaf infiltration with *Bp* PsJN inoculum (Bp LT) resulted in lower ΦPSI compared with Mock LT (**Figure 1A**). ΦPSI of *Bp* PsJN-inoculated leaves recovered thereafter. In all measurements, no significant modification of Y(ND) and Y(NA) was observed after leaf infiltration with *Bp* PsJN. Similarly, root or seed inoculation by *Bp* PsJN (Bp-RT or ST, respectively) showed identical ΦPSI, Y(ND), and Y(NA) values compared with their respective Mock plants (**Figure 1A**). Injection of *Bp* PsJN in leaves temporarily triggered a decrease in ETRI at 1 dpi (Bp LT, **Figure 1B**; Supplementary Table S1). However, at 3 dpi, ETRI was restored to the initial level observed before infiltration (i.e., 0 dpi). Soil and seed inoculation with *Bp* PsJN did not modify ETRI (**Figure 1B**). The excitation energies absorbed by PSII centers are contributed in photochemical utilization (ΦPSII) or heat dissipation (Nelson and Yocum, 2006). In PSII, the heat dissipation includes regulated and non-regulated energy dissipation [Y(NPQ) and Y(NO), respectively]. At all time points, no significant difference was observed in ΦPSII, Y(NPQ), and Y(NO) in Bp-LT, -RT, or -ST compared with their respective control (**Figure 1C**). No modification in ETRII, as well as Fv/Fm, was due to *Bp* PsJN presence whatever inoculation mode (**Figures 1D,E**, respectively). Independently to PSII, PSI is capable of recycling electrons *via* cyclic electron flow (Shikanai, 2014). A lower Y(CEF), was observed only at 1 dpi in *Bp* PsJN-inoculated leaves compared with MgCl_2_-inoculated leaves (**Figure 1F**). *Bp* PsJN inoculation of seeds or by soil drenching did not modify Y(CEF) values. All these results indicated that *Bp* PsJN inoculation locally and temporarily restricts cyclic electron flow and photochemistry of PSI.

#### Gas Exchanges

**FIGURE 2:**
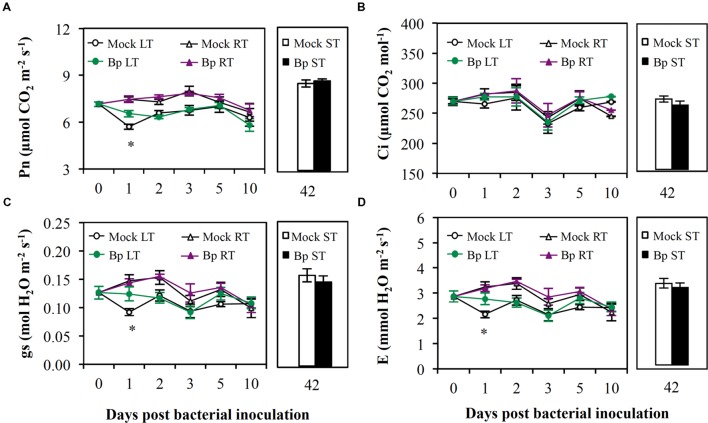
**Gas exchanges in mature leaves after LT, RT, or ST with *B. phytofirmans* strain PsJN.** Pn **(A)**, Ci **(B)**, gs **(C)**, and E **(D)** were analyzed. Data (mean ± SE) are averages of three experimental replicates, each with three plants per treatment (*n* = 9). *, #, or aaa, indicate significant differences in Bp-LT, -RT, or -ST conditions compared with their respective control, respectively (Student’s *t*-test; *P* < 0.05).

One day after leaf infiltration, Pn, gs, and E (**Figures 2A,C,D**, respectively) fell down in Mock LT plants (0 dpi; Supplementary Table S1) without modification of Ci (**Figure 2B**). This suggested that MgCl_2_ infiltration in leaves caused a non-stomatal limitation of Pn. The presence of *Bp* PsJN in leaves prevented this reduction in Pn, gs, and E (Bp LT; **Figures 2A,C,D**, respectively). Soil drenching or seed inoculation with *Bp* PsJN did not alter any parameters of gas exchange in *A. thaliana* plants (**Figure 2**).

### Modifications in Metabolite Contents by *Bp* PsJN Inoculation are Dependent on Both Bacterial Location and Presence Period

**FIGURE 3:**
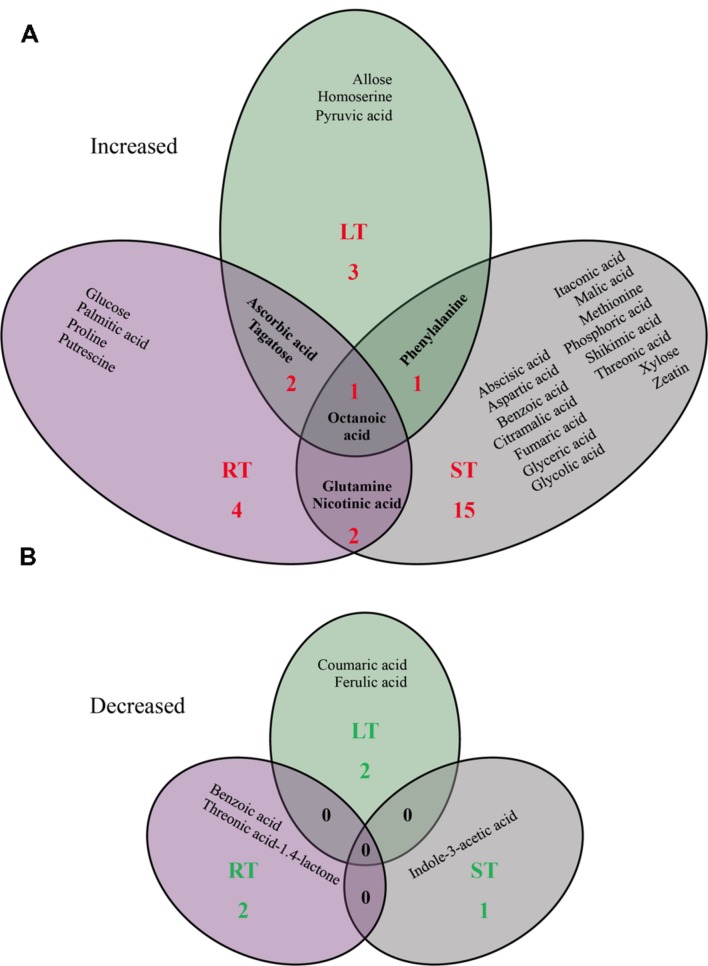
**Venn diagram of significant increase **(A)** or decrease **(B)** in leaf metabolite profiles triggered by LT, RT, or ST with *B. phytofirmans* strain PsJN (Student’s *t*-test; *P* ≤ 0.05)**.

Since metabolites are often the end products of regulatory mechanisms, the plant metabolome provides a wealth of information that helps to better understand plant responses to their environment (Feussner and Polle, 2015). We thus focused on plant metabolite profiling to reveal a snapshot of the physiological modifications in the leaves of *Bp* PsJN-inoculated plants. Considering different physicochemical properties of diverse metabolites, GC–MS, LC–MS, and UPLC-ESI-MS/MS analysis were performed. LC–MS is known as a technique more sensitive than GC–MS in metabolomic investigations, considering less extensive sample preparation such as chemical derivatization (Guérard et al., 2011). Among the 81 metabolites (72 by GC–MS, 5 by LC–MS, and 4 by UPLC-ESI-MS/MS) identified in all leaf samples, nine (seven increased and two decreased) metabolite contents were significantly different in Bp-LT leaves, 11 (nine increased and two decreased) between in Bp-RT leaves, and 20 (19 increased and 1 decreased) in Bp-ST leaves compared to their corresponding control (**Figure 3**; *P* < 0.05). The number of these changes is comparable with previous study showing that accumulation of 13 metabolites was affected in leaves of *P. fluorescens* SS101-treated plants (van de Mortel et al., 2012). Surprisingly, the majority of the observed changes was specific to one mode of bacterial inoculation, only one compound (octanoic acid) accumulation was displayed by the three bacterial inoculation modes, and no decrease in compound level was found in common of these three bacterial treatments.

#### Primary Metabolites

**FIGURE 4:**
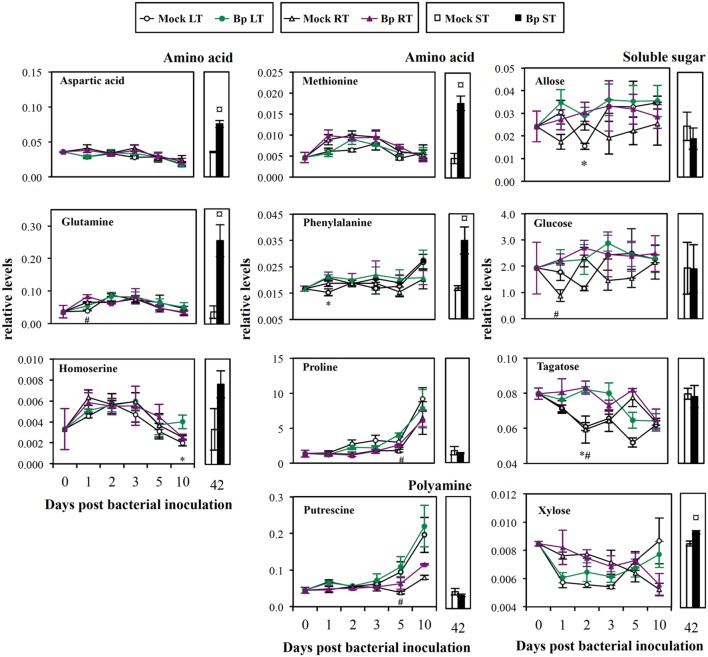
**Mature leaf amino acid, soluble sugar and polyamine levels after LT, RT, or ST with *B. phytofirmans* strain PsJN**. Relative concentration was expressed relatively to an internal standard (arbitrary unit). Data (mean ± SE) are averages of three independent experimental replicates, each with three plants per treatment (*n* = 3). *, #, or aaa, indicate significant differences in Bp-LT, -RT, or -ST conditions compared with their respective control, respectively (Student’s *t*-test; *P* ≤ 0.05).

**FIGURE 5:**
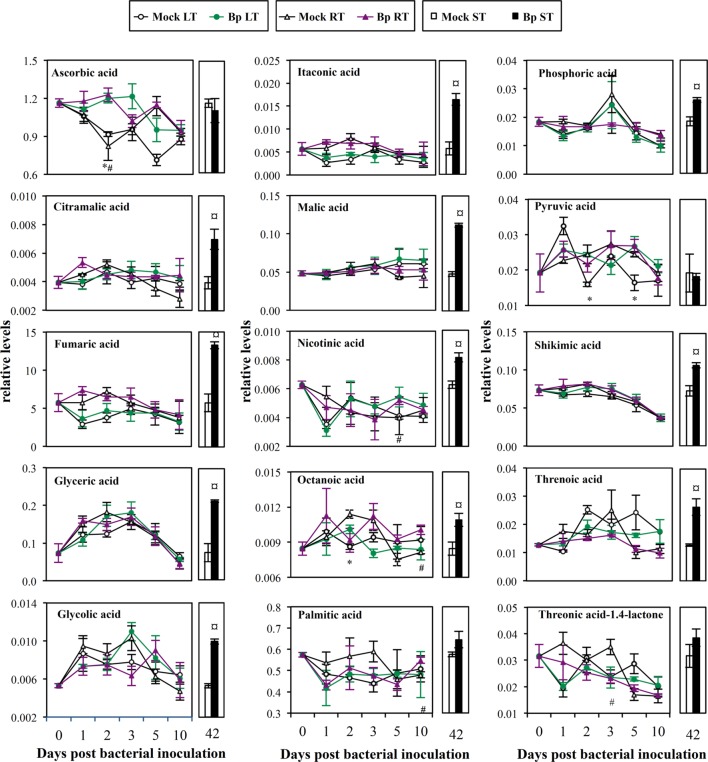
**Mature leaf organic acid levels after LT, RT, or ST with *B. phytofirmans* strain PsJN.** Relative concentration was expressed relatively to an internal standard (arbitrary unit). Data (mean ± SE) are averages of three independent experimental replicates, each with three plants per treatment (*n* = 3). *, #, or aaa, indicate significant differences in Bp-LT, -RT, or -ST conditions compared with their respective control, respectively (Student’s *t*-test; *P* < 0.05).

Compared with Mock LT leaves, leaf infiltration with *Bp* PsJN showed transitorily higher contents of amino acids (phenylalanine and homoserine, 1 and 10 dpi, respectively; **Figure 4**), soluble sugars (allose and tagatose, 2 dpi; **Figure 4**), vitamin C (ascorbic acid, 2 dpi; **Figure 5**), octanoic acid (2 dpi; **Figure 5**), and pyruvic acid (2 and 4 dpi, **Figure 5**; Supplementary Table S2).

Compared with Mock RT leaves, higher levels of amino acids (glutamine and proline, 1 and 5 dpi, respectively; **Figure 4**), soluble sugars (glucose and tagatose, 1 and 2 dpi, respectively; **Figure 4**), aliphatic polyamine (putrescine, 5 dpi; **Figure 4**), vitamins (ascorbic acid and nicotinic acid, 2 and 5 dpi, respectively; **Figure 5**) and saturated fatty acids (octanoic and palmitic acids, 10 dpi; **Figure 5**) were transitorily induced by inoculation of *Bp* PsJN by soil drenching (Supplementary Table S3). Moreover, a lower level of vitamin C derivative (L-threonic acid-1.4-lactone) was detected in *Bp* PsJN-inoculated plants in comparison to Mock RT plants at 2 dpi (**Figure 5**).

Seed inoculation with *Bp* PsJN led to the accumulation of 17 metabolites in *A. thaliana* leaves 42 dpi compared with Mock ST leaves (Supplementary Table S4), including amino acids (aspartic acid, glutamine, methionine, and phenylalanine; **Figure 4**), soluble sugar (xylose; **Figure 4**), intermediates or derivatives of TCA cycle (fumaric, malic, and itaconic acids, respectively; **Figure 5**), sugar acids (glyceric and threonic acids; **Figure 5**), vitamin B3 (nicotinic acid; **Figure 5**), precursor of the aromatic amino acid metabolism (shikimic acid) and citramalic, glycolic, octanoic, and phosphoric acids (**Figure 5**).

#### Secondary Metabolites

**FIGURE 6:**
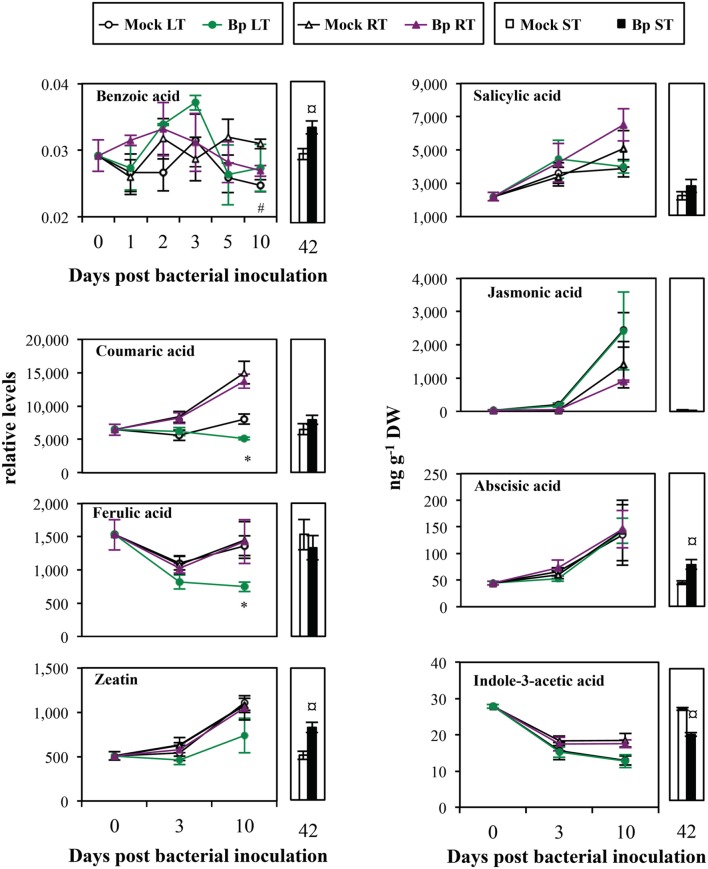
**Mature leaf secondary metabolite levels after LT, RT, or ST with *B. phytofirmans* strain PsJN.** Relative levels of benzoic, coumaric, ferulic acids, and zeatin were normalized by internal standard (arbitrary unit). Concentrations of salicylic acid, jasmonic acid, abscisic acid, and indole-3-acetic acid were expressed as ng per g dry weight of leaves (ng g^-1^ DW). Only benzoic acid was detected by GC–MS, the others metabolites were detected by UPLC-ESI-MS/MS. Data (mean ± SE) are averages of three experimental replicates, each with three plants per treatment (*n* = 3). *, #, or aaa, indicate significant differences in Bp-LT, -RT, or -ST conditions compared with their respective control, respectively (Student’s *t*-test; *P* < 0.05).

Benzoic acid content accumulated in leaves owing to seed inoculation with *Bp* PsJN (Bp ST, 42 dpi; **Figure 6**), whereas, it decreased in bacterized roots (Bp RT, 10 dpi; **Figure 6**). Moreover, lower coumaric and ferulic acid levels were observed in Bp LT leaves than in Mock LT leaves at 10 dpi (**Figure 6**).

After leaf or soil treatments, levels of hormones, salicylic acid, jasmonic acid, ABA, and zeatin increased with time in both Mock and Bp plants, except for IAA which decreased (**Figure 6**). Contents of salicylic acid and jasmonic acid were not affected by presence of *Bp* PsJN, independently of the inoculation method. Similarly, ABA, IAA, and zeatin levels were not modified by leaf infiltration or soil drenching with *Bp* PsJN. However, seed inoculation with *Bp* PsJN, which results in a relative long-term colonization in *A. thaliana*, led to an increase in ABA and zeatin contents, but a decrease in IAA level.

## Discussion

**FIGURE 7:**
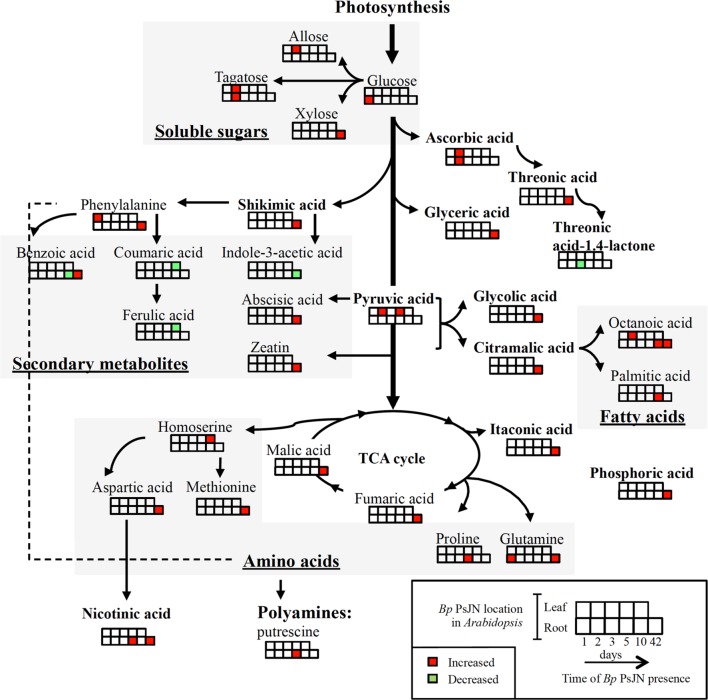
**Overview of the metabolic response in *Arabidopsis* leaf to LT, RT, or ST with *B. phytofirmans* strain PsJN.** Significant changes in bacterized plants compared to the corresponding mock plants are shown (Student’s *t*-test; *P* < 0.05).

Despite abundant researches on plant–PGPR interactions, PGPR-induced host plant responses were relatively less studied within a global view. Whereas, several investigations used transcriptomic or proteomic approaches, only few works performed metabolomic studies to assess this interaction (Walker et al., 2011; van de Mortel et al., 2012; Weston et al., 2012; Chamam et al., 2013; Couillerot et al., 2013). In this study, *Bp* PsJN-mediated modifications in metabolites of *A. thaliana* leaves were studied (**Figure 7**). Changes in those end products of regulatory processes get more insights the closest to the phenotype and could thus allow to better understand the interaction between *A. thaliana* and *Bp* PsJN.

### Only a Local Presence of *Bp* PsJN Modified PSI Activity

We showed that seed inoculation and soil drenching with *Bp* PsJN led to root endophytic colonization in *A. thaliana*, whereas leaf infiltration with *Bp* PsJN provoked leaf endophytic colonization. Although, PGPR rhizosphere and root colonization were known to result in leaf physiological modifications, perception and responses to PGPR leaf inoculation were less understood. Only few studies showed that leaf spraying with several PGPR strains (*Pseudomonas* sp. or *Bacillus* sp.) increased leaf nutrient levels and plant growth through the release of bacterial compounds, such as amino acids, vitamins, and hormones (Esitken et al., 2003; Dakora, 2015). In this study, we showed that *A. thaliana* leaf responses in photosynthetic performance as well as metabolite contents to *Bp* PsJN are dependent on time (days or weeks) and site (local or distal) of inoculation.

Presence of *Bp* PsJN in *A. thaliana* leaves did not affect PSII center activity. In contrast, PSI appeared to be susceptible to leaf infiltration with *Bp* PsJN but not with MgCl_2_ since ΦPSI, ETRI, and Y(CEF) were diminished by leaf infiltration with *Bp* PsJN 1 dpi. Photosynthetic production of chemical energy, adenosine triphosphate and reduced nicotinamide adenine dinucleotide phosphate, requires linear electron flow from PSII to PSI (Nelson and Yocum, 2006). This flow functions in series in thylakoid membranes of chloroplasts. Limitations of electron flows could lead to a restriction of the generation of adenosine triphosphate which is used in carbon assimilation (Shikanai, 2014). Furthermore, electrons can be recycled and driven solely by PSI (Shikanai, 2014). Y(CEF) allows to maintain high photosynthetic activity under variable environmental stress conditions *via* electron recycling by PSI (Sonoike, 2006; Shikanai, 2014). These results suggested that presence of *Bp* PsJN in leaves temporarily limited cyclic electron flow in PSI centers. To our knowledge, this is the first study showing PSI modifications induced by a PGPR inoculation. Moreover, PSI activity is known to be inhibited by accumulation of reactive oxygen species during abiotic stress (Sonoike, 2006). Recently, it was shown that *Bp* PsJN rapidly elicited H_2_O_2_ production in *A. thaliana* cell cultures (Trdá et al., 2014). This could explain the decrease in PSI activity few hours after *Bp* PsJN infiltration in leaves. Non-stomatal Pn limitation and decrease in gs and E were temporarily detected in Mock-infiltrated leaves 1 dpi. Consistently with previous data (Su et al., 2015), *Bp* PsJN inoculation by seed immersion or soil drenching, which only led to root colonization in *A. thaliana* plants, did not modify any parameters of photosynthesis. However, presence of *Bp* PsJN in leaves attenuated Pn limitation and stomatal closure. Alterations of photosynthesis by *Bp* PsJN were observed in maize (Naveed et al., 2014) and grapevine plantlets (Ait Barka et al., 2006; Fernandez et al., 2012). Additionally, *Bp* PsJN is capable to migrate to leaves of the maize and the grapevine plantlets following root or seed inoculation, respectively (Compant et al., 2008; Naveed et al., 2012). All these results suggested that modification of photosynthetic activity could require *Bp* PsJN presence in leaves.

### Accumulation of Soluble Sugars in Response to PGPR Inoculation

Higher photosynthetic products such as soluble sugars (allose and tagatose, LT Bp, 2 dpi; glucose and tagatose, RT Bp, 1 or 2 dpi and xylose, ST Bp, 42 dpi) were detected in bacterized plants. Allose and tagatose were epimer of glucose and fructose, respectively. Soluble sugars may derive from the mobilization of carbon reserves stored in starch and/or from precursor accumulations (such as galactinol, phosphated, and nucleotide sugars). *A. thaliana* root inoculation with *P. fluorescens* GM30 or Pf-5 stimulated glucose and fructose contents in leaves (Weston et al., 2012). Higher soluble sugar contents, such as those of glucose, fructose, maltose, sucrose, raffinose and mannose, were found in leaves of *Bp* PsJN-inoculated grapevine plantlets 4 weeks after root inoculation (Fernandez et al., 2012). Moreover, soluble sugars are available nutrients, cell wall components (Brown et al., 2005) and transmissible signals of plant development, such as the onset of flowering (Bernier et al., 1993) and senescence (Rolland et al., 2002). Consistently, Poupin et al. (2013) showed early flowering and accelerated life cycle in *Bp* PsJN-treated *A. thaliana* plants.

### Manipulation of Polyamine, Amino Acid, or Vitamin Metabolism during Plant–PGPR Interaction

Polyamine putrescine and amino acid (aspartic acid, glutamine, homoserine, methionine, phenylalanine, and proline) contents were increased in *Bp* PsJN-inoculated plants. Similarly, PGPR strain *Azotobacter vinelandii* was capable to synthesize and liberate amino acids such as arginine, tryptophan, and methionine (Gonzalez-Lopez et al., 1983). *P. fluorescens* GM30 could elicit amino acid metabolism of *A. thaliana* shoot after root inoculation, shown by tryptophan and phenylalanine accumulations (Weston et al., 2012). Here, a larger variety of amino acids was stimulated by seed inoculation suggesting that amino acid accumulation induced by *Bp* PsJN was influenced by the time of bacterial presence. Polyamines and amino acids are required to increase yield and overall quality of crops, as well as to resist to stress conditions (Aziz et al., 1997; van Damme et al., 2009). Proline accumulation has been detected in *Bp* PsJN-inoculated grapevine plantlets (Ait Barka et al., 2006), but not in *A. thaliana* (Pinedo et al., 2015). Synthesis of proline, a well-known osmolyte, could be stimulated by osmotic stress, such as salinity or cold (Verslues and Sharma, 2010). Inoculation by *Bp* PsJN accelerated accumulation of proline after exposure of salinity (Pinedo et al., 2015) or chilling (Ait Barka et al., 2006) in *A. thaliana* or grapevine plantlets, respectively. The proline accumulation observed here could thus explain tolerance of *Bp* PsJN-inoculated *A. thaliana* plants toward cold stress (Su et al., 2015). Methionine is the precursor of ethylene. Regulation of ethylene biosynthesis is necessary to several plant physiological processes (germination, root nodulation, flowering, abscission, senescence, and programmed cell death) and defense responses (Wen, 2015). Phenylalanine is a precursor of secondary metabolites including flavonoids, various phenolic acids, and stilbenes. In this study, we also detected an accumulation of several phenolic acids in leaves of Bp ST plants, i.e., of long time *Bp* PsJN-root colonized plants. Shikimic acid is the precursor of the aromatic amino acid metabolism leading, for example, to the synthesis of flavonoid and phenylpropanoid compounds leading to lignin biosynthesis. Activity of a crucial enzyme in this process, phenylalanine ammonia lyase, was enhanced by *Bp* PsJN inoculation in potato plantlets, with increased phenolic amounts (Nowak et al., 1998) and lignin contents (Frommel et al., 1991). Here, lignin precursors, coumaric and ferulic acids, were decreased at 10 days after inoculation of *Bp* PsJN in *A. thaliana* leaves, which could be due to the acceleration of lignin synthesis. Lignin rigidifies and strengthens the cell wall structure through covalent cross-linkages to cell wall polysaccharides (Jung and Deetz, 1993). Consistent with this, a thicker mesophyll cell wall was observed in Bp ST plants (Su et al., 2015). Other organic acids detected in our samples were TCA cycle intermediates, such as fumaric and malic acids, or TCA cycle derivative such as itaconic acid. TCA cycle is in common with the catabolism of carbohydrates (glycolysis, pentose phosphate pathway), lipids (β-oxidation) and amino acids in the mitochondrial matrix (Hopkins, 2003). This pathway constitutes a key process in the delivery of carbon skeletons and energy for a wide array of biochemical reactions. Moreover, deficiency in expression of genes involved in the TCA cycle revealed metabolic variations that influence cell wall composition and accumulation (van der Merwe et al., 2010; Kusano et al., 2011). Moreover, fatty acids such as octanoic and palmitic acids play a major role in membrane/phospholipid biosynthesis.

Increase in vitamin C (ascorbic acid) and vitamin B3 (nicotinic acid) contents due to *Bp* PsJN colonization in leaf and/or root, respectively, was observed. Production of vitamins by PGPR, including vitamins C (Bremus et al., 2006) and B3 (Marek-Kozaczuk and Skorupska, 2001) was previously shown. Moreover, increase in carbohydrate levels could directly stimulate biosynthesis of vitamins produced either by plants (Smirnoff and Wheeler, 2000; Roje, 2007) or by bacteria (Roje, 2007). Vitamins C, B1 and B6 were shown to promote maize leaf growth (Stoianov, 1978). Vitamin C participates in diverse processes in plants, such as cell wall growth and cell expansion, photosynthesis and photoprotection, hormone synthesis (including ethylene and gibberellins), as well as resistance to abiotic stresses (Smirnoff and Wheeler, 2000). Vitamin B3 contributes to the production of cofactors in diverse cellular oxidation–reduction reactions (Kirkland, 2007) and has potentially protective role under stress conditions (Noctor et al., 2006).

### A Long-lasting Presence of *Bp* PsJN is Necessary for Modulating Hormonal Levels

A relatively long period (42 days) of *Bp* PsJN presence in root not only led to more modifications of primary metabolite profiling, but also of altered hormone levels in leaves. Increased ABA and zeatin (cytokinin) levels and decreased IAA (auxin) contents were found in *A. thaliana* 6 weeks after bacterization. Increased zeatin level in leaves by *Bp* PsJN inoculation is in accordance with the results of Lazarovits and Nowak (1997). *Bp* PsJN was capable to synthesize IAA (Weilharter et al., 2011), and this capacity was involved in *A. thaliana* growth promotion (Zuniga et al., 2013). Despite a decreased IAA level in leaves of *Bp* PsJN-inoculated plants, we showed that *Bp* PsJN endophytic colonization of *A. thaliana* roots increased leaf mass (Su et al., 2015). However, plant growth is dependent on antagonistic and/or synergistic interactions between all hormones rather than on the action of only one hormone. The antagonistic relationship between ABA and IAA is displayed at the biosynthesis level (Du et al., 2013). To maintain plant growth, auxin and cytokinin interact at the levels of biosynthesis, degradation, transport, and signaling to control the balance between the rate of cell division and differentiation (Schaller et al., 2015). Increased cytokinin contents could favor photosynthesis by promoting action on chloroplast ultrastructure, chlorophyll synthesis (Cortleven and Schmülling, 2015) and stomatal opening (Arkhipova et al., 2007). This could explain that stomatal closure observed in control treatment was avoided by the presence of *Bp* PsJN in leaves. Furthermore, *Azospirillum brasilense* (a PGPR strain producing ABA) increased ABA content of *A. thaliana* plants 3 weeks after inoculation (Cohen et al., 2008), followed by a higher photosynthetic pigment contents (Cohen et al., 2015). Consistently, increased photosynthetic pigment contents have been detected in *Bp* PsJN-seed inoculated *A. thaliana* plants (Su et al., 2015).

### Three Modes of Inoculation, Three Distinct Metabolite Profiles

It is well-known that perception of microorganisms by roots or by leaves differed (Balmer and Mauch-Mani, 2013; De Coninck et al., 2015). Some molecules display organ-specific inducibility. Significant differences were found in the gene expression profiles in challenged roots compared to those in challenged leaves (Badri et al., 2008). Moreover, different signaling pathways between roots and leaves are involved in *A. thaliana* defense responses (Millet et al., 2010). This difference in microorganism perception between roots and leaves, could partially explain the different metabolite response between root, seed, and leaf bacterial inoculation. Planchamp et al. (2014) showed that some ABA- and SA-dependent genes were regulated in roots inoculated with *Pseudomonas putida* KT2440, suggesting that this PGPR could influence hormonal accumulation in roots. Here, seed bacterization triggered root colonization and hormonal changes in leaves. Presence of the bacterium in roots after seed bacterization could thus trigger modifications in phytohormone levels in roots. These phytohormones could then spread to the leaves in order to induce other metabolite changes since plant hormones are important regulators of local and distal responses. However, the exact nature of such signaling molecule needs further investigation. Moreover, RT and ST plants displaying the same distribution of *Bp* PsJN, this suggested the existence of different signaling pathways that propagate to the distal part of the plant, depending on the time of presence of the PGPR.

In a previous study, we demonstrated that *Bp* PsJN inoculation, either by root or seed inoculation, stimulated *A. thaliana* growth (Su et al., 2015). Here, our results demonstrated that inoculation of *Bp* PsJN transitorily affected PSI activity. Since, metabolomic analysis is now admitted as one technology able to characterize plant–microbe interactions closest to the plant phenotype (Feussner and Polle, 2015), the metabolite data presented here allow us to speculate as to whether the changes in metabolite levels could revealed potential mechanisms involved in the induction of plant growth promotion and stress tolerance by *Bp* PsJN. Several amino acids accumulated in response to PsJN inoculation. Our data also showed that accumulation of octanoic acid is observed in bacterized plants independently of the inoculation method. In contrast, only seed inoculation triggered changes in TCA intermediate or derivative and in phytohormone contents, such as an increase in ABA and zeatin levels and a decrease in IAA contents. Thus, variation of a few compound contents in leaf can be elicited by the presence of *Bp* PsJN in leaves or by a distal impact of *Bp* PsJN root colonization, whereas, high magnitude of modifications was owing to a long period of bacterial presence in root.

## Author Contributions

FS, SD-C, and NV-G designed the research. FS, FG, FG, SC, NV-G, and SD-C carried out the experiments and analysis/interpretation of data. FS, CC, NV-G, and SD-C wrote the manuscript with contributions and discussion from all of the co-authors. All authors have given approval to the final version of the manuscript.

## Conflict of Interest Statement

The authors declare that the research was conducted in the absence of any commercial or financial relationships that could be construed as a potential conflict of interest.

## Abbreviations

*Bp* PsJN: *Burkholderia phytofirmans* strain PsJN
Ci: intercellular CO_2_ concentration
dpi: days post-bacterial inoculation
E: transpiration rate
ETR: electron transport rate
Fv/Fm: maximum photochemical efficiency of PSII
gs: stomatal conductance
PGPR: plant growth-promoting rhizobacteria
Pn: net photosynthesis
PS: photosystem
TCA: tricarboxylic acid
UPLC-ESI-MS/MS: ultra-performance liquid chromatography coupled with electrospray ionization/quadrupole-time-of-flight mass spectrometry
Y(CEF): quantum yield of cyclic electron flow
Y(NA): PSI acceptor side limitation
Y(ND): PSI donor side limitation
Y(NO): quantum yield of non-regulated energy dissipation in PSII
Y(NPQ): quantum yield of regulated energy dissipation in PSII
ΦPSI/II: effective quantum yield of PSI or II.

## References

[B1] Agrawal R. Satlewal A. Varma A. (2015). “Characterization of plant growth-promoting rhizobacteria (PGPR): a perspective of conventional versus recent techniques,” in *Heavy Metal Contamination of Soils*, eds Sherameti I. Varma A. (New York, NY: Springer International Publishing), 471–485.

[B2] Ait Barka E. Nowak J. Clément C. (2006). Enhancement of chilling resistance of inoculated grapevine plantlets with a plant growth-promoting rhizobacterium, *Burkholderia phytofirmans* strain PsJN. *Appl. Environ. Microbiol.* 72 7246–7252. 10.1128/AEM.01047-06 16980419 PMC1636148

[B3] Arkhipova T. Prinsen E. Veselov S. Martinenko E. Melentiev A. Kudoyarova G. (2007). Cytokinin producing bacteria enhance plant growth in drying soil. *Plant Soil* 292 305–315. 10.1007/s11104-007-9233-5

[B4] Aziz A. Martin-Tanguy J. Larher F. (1997). Plasticity of polyamine metabolism associated with high osmotic stress in rape leaf discs and with ethylene treatment. *Plant Growth Regul.* 21 153–163. 10.1023/A:1005730509433

[B5] Badri D. V. Loyola-Vargas V. M. Du J. Stermitz F. R. Broeckling C. D. Iglesias-Andreu L. (2008). Transcriptome analysis of *Arabidopsis* roots treated with signaling compounds: a focus on signal transduction, metabolic regulation and secretion. *New Phytol.* 179 209–223. 10.1111/j.1469-8137.2008.02458.x 18422893

[B6] Balmer D. Mauch-Mani B. (2013). More beneath the surface? Root versus shoot antifungal plant defenses. *Front. Plant Sci.* 4:256 10.3389/fpls.2013.00256 PMC3709096 23874350

[B7] Benhamou N. Kloepper J. W. Quadt-Hallman A. Tuzun S. (1996). Induction of defense-related ultrastructural modifications in pea root tissues inoculated with endophytic bacteria. *Plant Physiol.* 112 919–929. 12226427 10.1104/pp.112.3.919 PMC158019

[B8] Bernier G. Havelange A. E. Houssa C. Petitjean A. Lejeune P. (1993). Physiological signals that induce flowering. *Plant Cell* 5 1147–115. 10.1105/tpc.5.10.1147 12271018 PMC160348

[B9] Bolton M. D. (2009). Primary metabolism and plant defense-fuel for the fire. *Mol. Plant Microbe Interact.* 22 487–497. 10.1094/MPMI-22-5-0487 19348567

[B10] Boruah D. H. Kumar D. B. (2002). Plant disease suppression and growth promotion by a fluorescent *Pseudomonas* strain. *Folia Microbiol.* 47 137–143. 10.1007/BF02817671 12058391

[B11] Bremus C. Herrmann U. Bringer-Meyer S. Sahm H. (2006). The use of microorganisms in L-ascorbic acid production. *J. Biotechnol.* 124 196–205. 10.1016/j.jbiotec.2006.01.010 16516325

[B12] Brown D. M. Zeef L. A. Ellis J. Goodacre R. Turner S. R. (2005). Identification of novel genes in *Arabidopsis* involved in secondary cell wall formation using expression profiling and reverse genetics. *Plant Cell* 17 2281–2295. 10.1105/tpc.105.031542 15980264 PMC1182489

[B13] Chamam A. Sanguin H. Bellvert F. Meiffren G. Comte G. Wisniewski-Dyé F. (2013). Plant secondary metabolite profiling evidences strain-dependent effect in the *Azospirillum*–*Oryza sativa* association. *Phytochemistry* 87 65–77. 10.1016/j.phytochem.2012.11.009 23266268

[B14] Cohen A. C. Bottini R. Piccoli P. N. (2008). *Azospirillum brasilense* Sp 245 produces ABA in chemically-defined culture medium and increases ABA content in *Arabidopsis* plants. *Plant Growth Regul.* 54 97–103. 10.1007/s10725-007-9232-9

[B15] Cohen A. C. Bottini R. Pontin M. Berli F. J. Moreno D. Boccanlandro H. (2015). *Azospirillum brasilense* ameliorates the response of *Arabidopsis thaliana* to drought mainly via enhancement of ABA levels. *Physiol. Plant.* 153 79–90. 10.1111/ppl.12221 24796562

[B16] Cohen A. C. Travaglia C. N. Bottini R. Piccoli P. N. (2009). Participation of abscisic acid and gibberellins produced by endophytic *Azospirillum* in the alleviation of drought effects in maize. *Botany* 87 455–462. 10.1139/B09-023

[B17] Compant S. Kaplan H. Sessitsch A. Nowak J. Ait Barka E. Clément C. (2008). Endophytic colonization of *Vitis vinifera* L. by *Burkholderia phytofirmans* strain PsJN: from the rhizosphere to inflorescence tissues. *FEMS Microbiol. Ecol.* 63 84–93. 10.1111/j.1574-6941.2007.00410.x 18081592

[B18] Cortleven A. Schmülling T. (2015). Regulation of chloroplast development and function by cytokinin. *J. Exp. Bot.* 66 4999–5013. 10.1093/jxb/erv132 25873684

[B19] Couillerot O. Ramírez-Trujillo A. Walker V. Von Felten A. Jansa J. Maurhofer M. (2013). Comparison of prominent *Azospirillum* strains in *Azospirillum*–*Pseudomonas*–*Glomus* consortia for promotion of maize growth. *Appl. Microbiol. Biotechnol.* 97 4639–4649. 10.1007/s00253-012-4249-z 22805783

[B20] Dakora F. D. (2015). “Lumichrome: a bacterial signal molecule influencing plant growth,” in *Biological Nitrogen Fixation* Vol. 2 ed. De Bruijn F. J. (Hoboken, NJ: John Wiley & Sons), 389.

[B21] Davies P. J. (2010). *Plant Hormones: Biosynthesis, Signal Transduction, Action!* Dordrecht: Springer Science & Business Media.

[B22] Davies W. J. Jones H. G. (1991). *Abscisic Acid Physiology and Biochemistry.* Oxford: BIOS Scientific Publishers.

[B23] De Coninck B. Timmermans P. Vos C. Cammue B. P. A. Kazan K. (2015). What lies beneath: belowground defense strategies in plants. *Trends Plant Sci.* 20 91–101. 10.1016/j.tplants.2014.09.007 25307784

[B24] Du H. Wu N. Chang Y. Li X. Xiao J. Xiong L. (2013). Carotenoid deficiency impairs ABA and IAA biosynthesis and differentially affects drought and cold tolerance in rice. *Plant Mol. Biol.* 83 475–488. 10.1007/s11103-013-0103-7 23846670

[B25] Esitken A. Karlidag H. Ercisli S. Turan M. Sahin F. (2003). The effect of spraying a growth promoting bacterium on the yield, growth and nutrient element composition of leaves of apricot (*Prunus armeniaca* L. cv. Hacihaliloglu). *Crop Pasture Sci.* 54 377–380. 10.1071/AR02098

[B26] Fanucchi F. Alpi E. Olivieri S. Cannistraci C. V. Bachi A. Alpi A. (2012). Acclimation increases freezing stress response of *Arabidopsis thaliana* at proteome level. *Biochim. Biophys. Acta* 1824 813–825. 10.1016/j.bbapap.2012.03.015 22510494

[B27] Fernandez O. Theocharis A. Bordiec S. Feil R. Jacquens L. Clément C. (2012). *Burkholderia phytofirmans* PsJN acclimates grapevine to cold by modulating carbohydrate metabolism. *Am. Phytopathol. Soc.* 25 496–504. 10.1094/MPMI-09-11-0245 22409157

[B28] Feussner I. Polle A. (2015). What the transcriptome does not tell—proteomics and metabolomics are closer to the plants’ patho-phenotype. *Curr. Opin. Plant Biol.* 26 26–31. 10.1016/j.pbi.2015.05.023 26051215

[B29] Frommel M. I. Nowak J. Lazarovits G. (1991). Growth enhancement and developmental modifications of in vitro grown potato (*Solanum tuberosum* spp. tuberosum) as affected by a nonfluorescent *Pseudomonas* sp. *Plant Physiol.* 96 928–936. 10.1104/pp.96.3.928 16668277 PMC1080867

[B30] Gonzalez-Lopez J. Salmeron V. Moreno J. Ramos-Cormenzana A. (1983). Amino acids and vitamins produced by *Azotobacter vinelandii* ATCC 12837 in chemically-defined media and dialysed soil media. *Soil Biol. Biochem.* 15 711–713. 10.1016/0038-0717(83)90037-8

[B31] Guérard F. Pétriacq P. Gakière B. Tcherkez G. (2011). Liquid chromatography/time-of-flight mass spectrometry for the analysis of plant samples: a method for simultaneous screening of common cofactors or nucleotides and application to an engineered plant line. *Plant Physiol. Biochem.* 49 1117–1125. 10.1016/j.plaphy.2011.06.003 21723140

[B32] Harrington J. M. Duckworth O. W. Haselwandter K. (2015). The fate of siderophores: antagonistic environmental interactions in exudate-mediated micronutrient uptake. *Biometals* 28 461–472. 10.1007/s10534-015-9821-4 25619589

[B33] Hopkins W. (2003). *Physiologie Végétale.* Bruscelles: De Boeck Supérieur.

[B34] Huang W. Zhang S.-B. Cao K.-F. (2010). Stimulation of cyclic electron flow during recovery after chilling-induced photoinhibition of PSII. *Plant Cell Physiol.* 51 1922–1928. 10.1093/pcp/pcq144 20861006

[B35] Jung H. Deetz D. (1993). “Cell wall lignification and degradability,” in *Forage Cell Wall Structure and Digestibility*, eds Jung H. G. Buxton D. R. Hatfield R. D. Ralph J. (Madison, WI: American Society of Agronomy), 315–346.

[B36] Kim S. Lowman S. Hou G. Nowak J. Flinn B. Mei C. (2012). Growth promotion and colonization of switchgrass (*Panicum virgatum*) cv. Alamo by bacterial endophyte *Burkholderia phytofirmans* strain PsJN. *Biotechnol. Biofuels* 5 37 10.1186/1754-6834-5-37 PMC3462104 22647367

[B37] Kirkland J. B. (2007). “Niacin,” in *Handbook of Vitamins*, eds Zempleni J. Rucker R. B. Mccormick D. B. Suttie J. (New York, NY: Taylor & Francis Group), 192–232.

[B38] Kusano M. Tohge T. Fukushima A. Kobayashi M. Hayashi N. Otsuki H. (2011). Metabolomics reveals comprehensive reprogramming involving two independent metabolic responses of *Arabidopsis* to UV-B light. *Plant J.* 67 354–369. 10.1111/j.1365-313X.2011.04599.x 21466600

[B39] Lazarovits G. Nowak J. (1997). Rhizobacteria for improvement of plant growth and establishment. *HortScience* 32 188–192.

[B40] Le Roux C. Del Prete S. Boutet-Mercey S. Perreau F. Balagué C. Roby D. (2014). The hnRNP-Q protein LIF2 participates in the plant immune response. *PLoS ONE* 9:e99343 10.1371/journal.pone.0099343 PMC4051675 24914891

[B41] Li-Marchetti C. Le Bras C. Relion D. Citerne S. Huché-Thélier L. Sakr S. (2015). Genotypic differences in architectural and physiological responses to water restriction in rose bush. *Front. Plant Sci.* 6:355 10.3389/fpls.2015.00355 PMC4443023 26074929

[B42] Lo Piccolo S. Ferraro V. Alfonzo A. Settanni L. Ercolini D. Burruano S. (2010). Presence of endophytic bacteria in *Vitis vinifera* leaves as detected by fluorescence in situ hybridization. *Ann. Microbiol.* 60 161–167. 10.1007/s13213-010-0023-6

[B43] Marek-Kozaczuk M. Skorupska A. (2001). Production of B-group vitamins by plant growth-promoting *Pseudomonas fluorescens* strain 267 and the importance of vitamins in the colonization and nodulation of red clover. *Biol. Fertil. Soils* 33 146–151. 10.1007/s003740000304

[B44] Miller G. Huang I. J. Welkie G. Pushnik J. (1995). “Function of iron in plants with special emphasis on chloroplasts and photosynthetic activity,” in *Iron Nutrition in Soils and Plants*, ed. Abadia J. (Dordrecht: Springer), 19–28.

[B45] Millet Y. A. Danna C. H. Clay N. K. Songnuan W. Simon M. D. Werck-Reichhart D. (2010). Innate immune responses activated in *Arabidopsis* roots by microbe-associated molecular patterns. *Plant Cell* 22 973–990. 10.1105/tpc.109.069658 20348432 PMC2861455

[B46] Naveed M. Mitter B. Reichenauer T. G. Wieczorek K. Sessitsch A. (2014). Increased drought stress resilience of maize through endophytic colonization by *Burkholderia phytofirmans* PsJN and *Enterobacter* sp. FD17. *Environ. Exp. Bot.* 97 30–39. 10.1016/j.envexpbot.2013.09.014

[B47] Naveed M. Mitter B. Sessitsch A. Reichenauer T. (2012). “Endophytic colonization of *Burkholderia phytofirmans* strain PsJN induces drought-stress tolerance in maize,” in *I World Congress on the Use of Biostimulants in Agriculture*, eds Silva S. S. Brown P. Ponchet M. (Leuven: ISHS Acta Horticulturae 1009), 117–125.

[B48] Nelson N. Yocum C. F. (2006). Structure and function of photosystems I and II. *Annu. Rev. Plant Biol.* 57 521–565. 10.1146/annurev.arplant.57.032905.105350 16669773

[B49] Noctor G. Queval G. Gakière B. (2006). NAD (P) synthesis and pyridine nucleotide cycling in plants and their potential importance in stress conditions. *J. Exp. Bot.* 57 1603–1620. 10.1093/jxb/erj202 16714307

[B50] Nowak J. Asiedu S. K. Bensalim S. Richards J. Stewart A. Smith C. (1998). From laboratory to applications: challenges and progress with in vitro dual cultures of potato and beneficial bacteria. *Plant Cell Tissue Organ Cult.* 52 97–103. 10.1023/a:1005965822698

[B51] Pfündel E. Klughammer C. Schreiber U. (2008). Monitoring the effects of reduced PS II antenna size on quantum yields of photosystems I and II using the Dual-PAM-100 measuring system. *PAM Appl. Notes* 1 21–24.

[B52] Pillay V. K. Nowak J. (1997). Inoculum density, temperature, and genotype effects on in vitro growth promotion and epiphytic and endophytic colonization of tomato (*Lycopersicon esculentum* L.) seedlings inoculated with a pseudomonad bacterium. *Can. J. Microbiol.* 43 354–361. 10.1139/m97-049

[B53] Pinedo I. Ledger T. Greve M. Poupin M. J. (2015). *Burkholderia phytofirmans* PsJN induces long-term metabolic and transcriptional changes involved in *Arabidopsis thaliana* salt tolerance. *Front. Plant Sci.* 6:466 10.3389/fpls.2015.00466 PMC4477060 26157451

[B54] Planchamp C. Glauser G. Mauch-Mani B. (2014). Root inoculation with *Pseudomonas putida* KT2440 induces transcriptional and metabolic changes and systemic resistance in maize plants. *Front. Plant Sci.* 5:719 10.3389/fpls.2014.00719 PMC4292437 25628626

[B55] Poupin J. M. Timmermann T. Vega A. Zuniga A. Gonzalez B. (2013). Effects of the plant growth-promoting bacterium *Burkholderia phytofirmans* PsJN throughout the life cycle of *Arabidopsis thaliana*. *PLoS ONE* 8:e69435 10.1371/journal.pone.0069435 PMC3711820 23869243

[B56] Rincón A. Valladares F. Gimeno T. E. Pueyo J. J. (2008). Water stress responses of two Mediterranean tree species influenced by native soil microorganisms and inoculation with a plant growth promoting rhizobacterium. *Tree Physiol.* 28 1693–1701. 10.1093/treephys/28.11.1693 18765374

[B57] Roje S. (2007). Vitamin B biosynthesis in plants. *Phytochemistry* 68 1904–1921. 10.1016/j.phytochem.2007.03.038 17512961

[B58] Rolland F. Moore B. Sheen J. (2002). Sugar sensing and signaling in plants. *Plant Cell* 14 S185–S205. 10.1105/tpc.010455 12045277 PMC151255

[B59] Saeed A. I. Sharov V. White J. Li J. Liang W. Bhagabati N. (2003). TM4: a free, open-source system for microarray data management and analysis. *Biotechniques* 34 374–378. 12613259 10.2144/03342mt01

[B60] Schaller G. E. Bishopp A. Kieber J. J. (2015). The yin-yang of hormones: cytokinin and auxin interactions in plant development. *Plant Cell* 27 44–63. 10.1105/tpc.114.133595 25604447 PMC4330578

[B61] Sessitsch A. Coenye T. Sturz A. V. Vandamme P. Ait Barka E. Salles J. F. (2005). *Burkholderia phytofirmans* sp. nov., a novel plant-associated bacterium with plant-beneficial properties. *Int. J. Syst. Evol. Microbiol.* 55 1187–1192. 10.1099/ijs.0.63149-0 15879253

[B62] Shikanai T. (2014). Central role of cyclic electron transport around photosystem I in the regulation of photosynthesis. *Curr. Opin. Biotechnol.* 26 25–30. 10.1016/j.copbio.2013.08.012 24679254

[B63] Smirnoff N. Wheeler G. L. (2000). Ascorbic acid in plants: biosynthesis and function. *Crit. Rev. Biochem. Mol. Biol.* 35 291–314. 10.1080/10409230008984166 11005203

[B64] Sonoike K. (2006). “Photoinhibition and protection of photosystem I,” in *Photosystem I*, ed. Golbeck J. H. (Dordrecht: Springer), 657–668.

[B65] Stoianov I. (1978). Influence of magnesium and of certain vitamins on the restoration of maize plants. *Dokl. Bolgarshoi Akad. Nauk* 31 457–460.

[B66] Su F. Jacquard C. Villaume S. Michel J. Rabenoelina F. Clément C. (2015). *Burkholderia phytofirmans* PsJN reduces impact to freezing temperatures on photosynthesis in *Arabidopsis thaliana*. *Front. Plant Sci.* 6:810 10.3389/fpls.2015.00810 PMC4591482 26483823

[B67] Tcherkez G. Mahé A. Gauthier P. Mauve C. Gout E. Bligny R. (2009). In folio respiratory fluxomics revealed by 13C isotopic labeling and H/D isotope effects highlight the noncyclic nature of the tricarboxylic acid “cycle” in illuminated leaves. *Plant Physiol.* 151 620–630. 10.1104/pp.109.142976 19675152 PMC2754646

[B68] Trdá L. Fernandez O. Boutrot F. Héloir M. C. Kelloniemi J. Daire X. (2014). The grapevine flagellin receptor VvFLS2 differentially recognizes flagellin-derived epitopes from the endophytic growth-promoting bacterium *Burkholderia phytofirmans* and plant pathogenic bacteria. *New Phytol.* 201 1371–1384. 10.1111/nph.12592 24491115

[B69] van Damme M. Zeilmaker T. Elberse J. Andel A. De Sain-Van Der Velden M. Van Den Ackerveken G. (2009). Downy mildew resistance in *Arabidopsis* by mutation of HOMOSERINE KINASE. *Plant Cell* 21 2179–2189. 10.1105/tpc.109.066811 19622802 PMC2729605

[B70] van de Mortel J. E. De Vos R. C. Dekkers E. Pineda A. Guillod L. Bouwmeester K. (2012). Metabolic and transcriptomic changes induced in *Arabidopsis* by the rhizobacterium *Pseudomonas fluorescens* SS101. *Plant Physiol.* 160 2173–2188. 10.1104/pp.112.207324 23073694 PMC3510139

[B71] van der Merwe M. J. Osorio S. Araújo W. L. Balbo I. Nunes-Nesi A. Maximova E. (2010). Tricarboxylic acid cycle activity regulates tomato root growth via effects on secondary cell wall production. *Plant Physiol.* 153 611–621. 10.1104/pp.109.149047 20118274 PMC2879791

[B72] Van Hulten M. Ton J. Pieterse C. M. Van Wees S. C. (2010). “Plant defense signaling from the underground primes aboveground defenses to confer enhanced resistance in a cost-efficient manner,” in *Plant Communication from an Ecological Perspective*, eds Baluska F. Ninkovic V. (Heidelberg: Springer), 43–60.

[B73] Verslues P. E. Sharma S. (2010). Proline metabolism and its implications for plant-environment interaction. *Arabidopsis Book* 8:e0140 10.1199/tab.0140 PMC3244962 22303265

[B74] von Caemmerer S. Farquhar G. D. (1981). Some relationships between the biochemistry of photosynthesis and the gas exchange of leaves. *Planta* 153 376–387. 10.1007/bf00384257 24276943

[B75] Walker V. Bertrand C. Bellvert F. Moënne-Loccoz Y. Bally R. Comte G. (2011). Host plant secondary metabolite profiling shows a complex, strain-dependent response of maize to plant growth-promoting rhizobacteria of the genus *Azospirillum*. *New Phytol.* 189 494–506. 10.1111/j.1469-8137.2010.03484.x 20946131

[B76] Weckwerth W. Wenzel K. Fiehn O. (2004). Process for the integrated extraction, identification and quantification of metabolites, proteins and RNA to reveal their co-regulation in biochemical networks. *Proteomics* 4 78–83. 10.1002/pmic.200200500 14730673

[B77] Weilharter A. Mitter B. Shin M. V. Chain P. S. Nowak J. Sessitsch A. (2011). Complete genome sequence of the plant growth-promoting endophyte *Burkholderia phytofirmans* strain PsJN. *J. Bacteriol.* 193 3383–3384. 10.1128/jb.05055-11 21551308 PMC3133278

[B78] Wen C.-K. (2015). *Ethylene in Plants.* Dordrecht: Springer.

[B79] Weston D. J. Pelletier D. A. Morrell-Falvey J. L. Tschaplinski T. J. Jawdy S. S. Lu T.-Y. (2012). *Pseudomonas fluorescens* induces strain-dependent and strain-independent host plant responses in defense networks, primary metabolism, photosynthesis, and fitness. *Mol. Plant Microbe Interact.* 25 765–778. 10.1094/MPMI-09-11-0253 22375709

[B80] Yadav B. K. Akhtar M. S. Panwar J. (2015). “Rhizospheric plant-microbe interactions: key factors to soil fertility and plant nutrition,” in *Plant Microbes Symbiosis: Applied Facets*, ed. Arora N. K. (New Delhi: Springer), 127–145.

[B81] Zhang H. Xie X. T. Kim M. S. Kornyeyev D. A. Holaday S. Paré P. W. (2008). Soil bacteria augment *Arabidopsis* photosynthesis by decreasing glucose sensing and abscisic acid levels in planta. *Plant J.* 56 264–273. 10.1111/j.1365-313X.2008.03593.x 18573192

[B82] Zhou X. Smaill S. J. Clinton P. W. (2013). Methane oxidation needs less stressed plants. *Trends Plant Sci.* 18 657–659. 10.1016/j.tplants.2013.09.011 24161402

[B83] Zuniga A. Poupin M. J. Donoso R. Ledger T. Guiliani N. Gutiérrez R. A. (2013). Quorum sensing and 3-indole acetic acid degradation play a role in colonization and plant growth promotion of *Arabidopsis thaliana* by *Burkholderia phytofirmans* PsJN. *Mol. Plant Microbe Interact.* 26 546–553. 10.1094/MPMI-10-12-0241-R 23301615

