# Supplementary material for: Spatio-temporal Responses of Arabidopsis Leaves in Photosynthetic Performance and Metabolite Contents to Burkholderia phytofirmans PsJN
Source: Front Plant Sci. 2016 Mar 30;7:403. doi: 10.3389/fpls.2016.00403 (PMC4811906; doi:10.3389/fpls.2016.00403)
Supplement: Supplementary file 1 [file Presentation_1.PDF]

Supplementary Material

Arabidopsis Leaf Responses in Photosynthetic Performance and Metabolite Contents to *Burkholderia phytofirmans* PsJN are Dependent on Bacterial Time of Presence or Location

Fan Su[1], Françoise Gilard[2], Florence Guérard[2], Sylvie Citerne[3], Christophe Clément[1], Nathalie Vaillant-Gaveau[1#], Sandrine Dhondt-Cordelier[1#*].

[1] Unité de Recherche Vignes et Vins de Champagne - EA 4707, SFR Condorcet FR CNRS 3417, UFR Sciences Exactes et Naturelles, Université de Reims Champagne-Ardenne, F-51687 Reims, France.
[2] Université Paris-Sud, Institute of Plant Sciences Paris-Saclay IPS2 (Bâtiment 630), UMR CNRS-INRA 9213, Saclay Plant Sciences, F-91405 Orsay, France.
[3] Institut Jean-Pierre Bourgin, UMR1318 INRA-AgroParisTech, ERL3559 CNRS, INRA Versailles-Grignon, F-78026 Versailles, France.

[#] NV-G and SD-C contributed equally to this work.

[*] **Correspondence:**
Sandrine Dhondt-Cordelier
sandrine.cordelier@univ-reims.fr

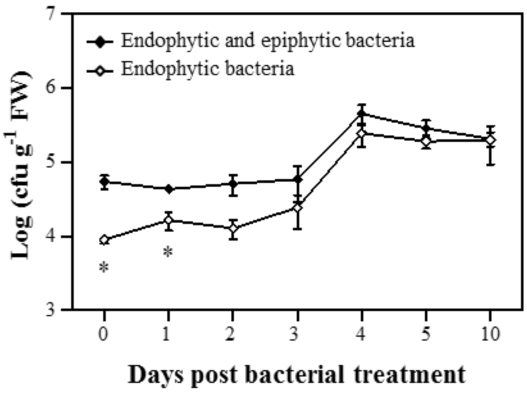

Supplemental Figure S1. Colonization of *Burkholderia phytofirmans* PsJN in *Arabidopsis* leaves. After leaf infiltration with *Bp* PsJN inoculum, numbers of bacteria in non-sterile (endophytic and epiphytic bacteria) or surface sterile (endophytic bacteria) *Arabidopsis* leaves (Bp LT) were expressed as log (cfu g^{-1} FW). Data (mean ± SE) are averages of 3 experimental replicates, each with 3 plants per treatment (n=3). Asterisks indicate significant differences between non sterile and surface sterile leaves in the same day (Student's *t* test; P < 0.05).

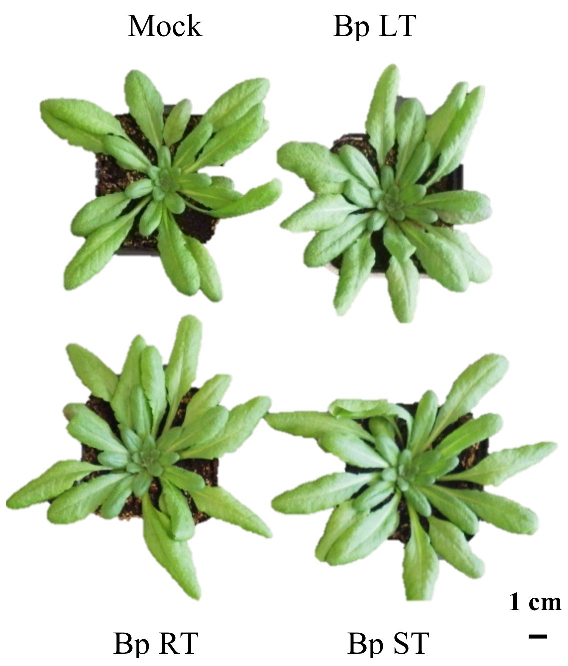

Supplemental Figure S2. Plant phenotype after colonization of *Burkholderia phytofirmans* PsJN. Photographs were taken 10 days after leaf infiltration with *Bp* PsJN inoculum (Bp LT), soil drenching (Bp RT) or 42 days after seed inoculation (Bp ST).

Supplemental Table S1. Leaf infiltration with or without *Burkholderia phytofirmans* PsJN decreased photosynthesis parameters compared with non-infiltrated plants (control) results from the Student's *t* test (P < 0.05).

	**1 dpi**		**2 dpi**		**3 dpi**		**5 dpi**		**10 dpi**	
	**Mock LT**	**Bp LT**	**Mock LT**	**Bp LT**	**Mock LT**	**Bp LT**	**Mock LT**	**Bp LT**	**Mock LT**	**Bp LT**
ETRI	P=0.925	P=0.018*	P=0.048*	P=0.045*	P=0.897	P=0.604	P=0.986	P=0.215	P=0.645	P=0.539
ETRII	P=0.128	P=0.120	P=0.021*	P=0.011*	P=0.645	P=0.741	P=0.154	P=0.513	P=0.645	P=0.523
Pn	P<0.001*	P=0.046*	P=0.037*	P=0.01*	P=0.186	P=0.181	P=0.576	P=0.753	P=0.003*	P=0.025*
Ci	P=0.576	P=0.417	P=0.559	P=0.426	P=0.115	P=0.057	P=0.226	P=0.874	P=0.902	P=0.396
gs	P=0.014*	P=0.905	P=0.743	P=0.555	P=0.062	P=0.057	P=0.113	P=0.918	P=0.198	P=0.261
E	P=0.019*	P=0.757	P=0.624	P=0.422	P=0.041*	P=0.027*	P=0.092	P=0.833	P=0.149	P=0.185

dpi, days post (bacterial) inoculation.

Supplemental Table S2. Metabolite profiling in *Arabidopsis thaliana* leaves after leaf infiltration with 10 mM $MgCl_2$ or *Burkholderia phytofirmans* PsJN solution

	0 dpi		1 dpi					2 dpi					3 dpi					5 dpi					10 dpi				
	Control		Mock LT		Bp LT			Mock LT		Bp LT			Mock LT		Bp LT			Mock LT		Bp LT			Mock LT		Bp LT		
	FC	SE	FC	SE	FC	SE	p	FC	SE	FC	SE	p	FC	SE	FC	SE	p	FC	SE	FC	SE	p	FC	SE	FC	SE	p
**Detected by GC-MS (relative level)**																											
Alanine	2.254	1.233	4.010	0.633	4.132	0.929	0.92	3.514	0.322	3.529	0.212	0.97	3.470	0.205	3.606	0.071	0.56	3.450	0.675	4.183	0.384	0.40	3.318	0.334	3.338	0.862	0.98
Arabinose	0.098	0.033	0.117	0.022	0.105	0.028	0.76	0.170	0.005	0.152	0.040	0.70	0.098	0.021	0.116	0.036	0.69	0.104	0.026	0.112	0.034	0.86	0.079	0.021	0.096	0.005	0.45
Ascorbic acid	1.161	0.034	1.059	0.043	1.115	0.048	0.44	0.915	0.017	1.201	0.037	0.89	0.955	0.043	1.215	0.098	0.07	0.713	0.045	0.949	0.093	0.08	0.878	0.045	0.946	0.046	0.35
Aspartic acid	0.036	0.001	0.029	0.002	0.029	0.003	0.98	0.033	0.004	0.034	0.005	0.10	0.028	0.002	0.033	0.004	0.35	0.028	0.007	0.029	0.006	0.97	0.019	0.003	0.017	0.003	0.81
Benzoic acid	0.029	0.002	0.027	0.003	0.027	0.003	0.87	0.027	0.003	0.034	0.000	0.12	0.031	0.004	0.037	0.001	0.24	0.026	0.002	0.026	0.005	0.93	0.025	0.001	0.027	0.004	0.50
Beta-Sitosterol	0.037	0.003	0.030	0.003	0.027	0.004	0.59	0.028	0.004	0.030	0.001	0.56	0.029	0.001	0.031	0.002	0.53	0.028	0.000	0.029	0.003	0.95	0.028	0.001	0.030	0.001	0.20
Citramalic acid	0.004	0.000	0.004	0.000	0.004	0.001	0.76	0.005	0.001	0.005	0.000	0.87	0.004	0.000	0.005	0.001	0.33	0.004	0.001	0.005	0.001	0.70	0.004	0.000	0.004	0.001	0.70
Citric acid	0.007	0.001	0.007	0.001	0.008	0.001	0.82	0.007	0.001	0.008	0.001	0.64	0.009	0.001	0.010	0.003	0.65	0.008	0.003	0.008	0.001	0.98	0.006	0.001	0.006	0.001	0.87
Cyclohexylamine	0.046	0.003	0.035	0.006	0.027	0.002	0.34	0.031	0.006	0.029	0.002	0.86	0.024	0.001	0.027	0.005	0.60	0.033	0.005	0.038	0.008	0.68	0.037	0.008	0.024	0.004	0.19
Allose	0.024	0.007	0.030	0.005	0.035	0.006	0.61	0.015	0.001	0.029	0.004	0.03	0.033	0.003	0.036	0.007	0.71	0.033	0.011	0.035	0.007	0.86	0.035	0.003	0.035	0.007	0.93
Digalactosylglycerol	0.067	0.011	0.023	0.004	0.024	0.005	0.96	0.027	0.002	0.030	0.008	0.72	0.039	0.006	0.046	0.001	0.34	0.066	0.013	0.055	0.007	0.53	0.075	0.002	0.072	0.007	0.77
Aminocaproic acid	0.017	0.000	0.017	0.000	0.017	0.000	0.73	0.018	0.000	0.017	0.001	0.65	0.016	0.000	0.016	0.000	0.94	0.017	0.001	0.016	0.000	0.80	0.017	0.000	0.016	0.000	0.80
Ethanolamine	1.825	0.355	1.250	0.289	1.714	0.245	0.29	0.601	0.058	1.411	0.483	0.23	1.546	0.216	1.894	0.095	0.21	1.549	0.565	1.489	0.304	0.93	1.290	0.363	1.027	0.256	0.59
Fructose	1.131	0.419	1.488	0.274	1.627	0.285	0.74	0.688	0.067	1.417	0.289	0.07	1.585	0.186	1.806	0.385	0.63	1.556	0.548	1.727	0.386	0.81	1.697	0.183	1.752	0.345	0.90
Fumaric acid	5.756	1.169	2.912	0.579	3.677	1.112	0.57	3.784	0.616	4.704	0.941	0.46	4.947	0.734	4.388	1.079	0.69	4.190	1.383	4.366	0.631	0.91	3.131	0.976	3.248	1.116	0.94
Galactinol	0.062	0.003	0.053	0.000	0.050	0.004	0.54	0.050	0.001	0.054	0.002	0.14	0.050	0.005	0.056	0.002	0.32	0.060	0.010	0.055	0.005	0.67	0.067	0.005	0.069	0.005	0.86
Galactonic acid	0.004	0.000	0.003	0.000	0.003	0.000	0.41	0.004	0.000	0.003	0.000	0.39	0.003	0.000	0.003	0.000	0.45	0.003	0.000	0.003	0.000	0.71	0.003	0.000	0.003	0.000	0.07
Galactose	0.010	0.000	0.008	0.000	0.009	0.001	0.14	0.008	0.001	0.009	0.001	0.32	0.009	0.000	0.010	0.001	0.54	0.011	0.002	0.011	0.001	0.86	0.015	0.002	0.016	0.001	0.98
Galactosylglycerol	0.070	0.017	0.044	0.005	0.046	0.011	0.86	0.034	0.002	0.048	0.016	0.47	0.043	0.006	0.064	0.006	0.07	0.066	0.017	0.057	0.007	0.65	0.083	0.008	0.076	0.009	0.56
Glucose 1	1.926	0.987	1.772	0.317	2.183	0.450	0.50	1.159	0.087	2.251	0.568	0.19	2.424	0.152	2.873	0.302	0.25	2.484	0.944	2.440	0.450	0.97	2.274	0.152	2.271	0.517	1.00
Glutamic acid	0.099	0.006	0.052	0.010	0.070	0.015	0.37	0.067	0.002	0.077	0.011	0.41	0.063	0.011	0.071	0.017	0.72	0.051	0.011	0.050	0.006	0.94	0.036	0.004	0.036	0.007	0.96
Glutamine	0.036	0.019	0.039	0.000	0.055	0.011	0.30	0.087	0.004	0.083	0.013	0.77	0.074	0.010	0.082	0.016	0.68	0.064	0.023	0.064	0.013	0.98	0.046	0.007	0.051	0.013	0.76
Glyceric acid	0.073	0.025	0.123	0.020	0.108	0.016	0.61	0.125	0.008	0.172	0.028	0.18	0.157	0.008	0.181	0.028	0.46	0.124	0.028	0.119	0.010	0.90	0.065	0.009	0.056	0.005	0.48
Glycerol	0.087	0.007	0.066	0.004	0.080	0.011	0.30	0.046	0.005	0.076	0.019	0.19	0.073	0.006	0.090	0.007	0.13	0.077	0.021	0.072	0.009	0.84	0.081	0.011	0.068	0.012	0.46
Glycine	0.046	0.025	0.252	0.046	0.319	0.069	0.47	0.211	0.058	0.199	0.012	0.85	0.147	0.015	0.181	0.055	0.59	0.093	0.035	0.132	0.025	0.40	0.119	0.012	0.104	0.025	0.61
Glycolic acid	0.005	0.000	0.009	0.002	0.007	0.000	0.55	0.008	0.001	0.008	0.001	0.99	0.008	0.001	0.011	0.001	0.06	0.007	0.001	0.008	0.002	0.59	0.006	0.001	0.006	0.001	0.82
Homoserine	0.003	0.002	0.005	0.000	0.005	0.000	0.09	0.006	0.001	0.006	0.001	0.90	0.005	0.001	0.006	0.001	0.28	0.003	0.001	0.004	0.001	0.62	0.002	0.000	0.004	0.001	0.05
IsoLeucine	0.073	0.013	0.055	0.004	0.079	0.022	0.33	0.063	0.008	0.063	0.004	0.99	0.063	0.002	0.068	0.003	0.28	0.073	0.007	0.073	0.013	1.00	0.171	0.033	0.174	0.029	0.95
Itaconic acid	0.006	0.001	0.003	0.001	0.004	0.001	0.41	0.003	0.001	0.005	0.000	0.36	0.006	0.001	0.004	0.001	0.30	0.003	0.001	0.004	0.001	0.45	0.003	0.001	0.003	0.001	0.70
Lactic acid	0.029	0.007	0.032	0.005	0.028	0.003	0.55	0.049	0.023	0.028	0.002	0.46	0.021	0.003	0.030	0.003	0.07	0.024	0.006	0.032	0.008	0.51	0.034	0.005	0.032	0.005	0.76

	0 dpi		1 dpi					2 dpi					3 dpi					5 dpi					10 dpi				
	Control		Mock LT		Bp LT			Mock LT		Bp LT			Mock LT		Bp LT			Mock LT		Bp LT			Mock LT		Bp LT		
	FC	SE	FC	SE	FC	SE	p	FC	SE	FC	SE	p	FC	SE	FC	SE	p	FC	SE	FC	SE	p	FC	SE	FC	SE	p
**Detected by GC-MS (relative level)**																											
Lauric acid	0.004	0.001	0.005	0.001	0.004	0.000	0.50	0.005	0.001	0.005	0.001	0.98	0.004	0.001	0.004	0.000	0.75	0.005	0.001	0.006	0.000	0.87	0.005	0.001	0.006	0.002	0.67
Leucine	0.047	0.011	0.029	0.003	0.049	0.017	0.31	0.034	0.007	0.035	0.003	0.90	0.034	0.001	0.036	0.003	0.62	0.046	0.007	0.044	0.010	0.91	0.129	0.027	0.131	0.023	0.95
Levoglucosan	0.061	0.028	0.074	0.019	0.059	0.022	0.63	0.091	0.005	0.087	0.030	0.90	0.049	0.014	0.057	0.024	0.80	0.053	0.022	0.065	0.029	0.76	0.037	0.017	0.046	0.002	0.65
Threitol	0.005	0.000	0.006	0.000	0.005	0.000	0.30	0.005	0.000	0.005	0.000	0.85	0.004	0.000	0.005	0.000	0.09	0.004	0.000	0.005	0.001	0.31	0.005	0.000	0.004	0.001	0.17
Lysine	0.013	0.003	0.006	0.000	0.010	0.003	0.32	0.009	0.001	0.009	0.001	0.90	0.009	0.000	0.010	0.001	0.44	0.010	0.002	0.010	0.003	0.97	0.016	0.002	0.018	0.003	0.68
Malic acid	0.048	0.003	0.045	0.004	0.047	0.007	0.84	0.049	0.004	0.057	0.006	0.36	0.053	0.005	0.058	0.012	0.74	0.061	0.019	0.067	0.014	0.81	0.061	0.006	0.065	0.015	0.79
Mannose	0.006	0.002	0.005	0.000	0.005	0.001	0.95	0.006	0.001	0.008	0.001	0.21	0.008	0.001	0.008	0.001	0.98	0.011	0.003	0.010	0.001	0.76	0.013	0.001	0.014	0.001	0.45
Melibiose	0.002	0.001	0.001	0.000	0.001	0.000	0.23	0.001	0.000	0.002	0.000	0.21	0.001	0.000	0.001	0.000	0.55	0.003	0.001	0.002	0.000	0.29	0.004	0.000	0.003	0.001	0.81
Methionine	0.005	0.001	0.006	0.001	0.006	0.001	0.68	0.006	0.000	0.009	0.001	0.12	0.008	0.002	0.008	0.000	0.84	0.004	0.000	0.006	0.001	0.43	0.006	0.002	0.006	0.001	0.98
Myo-inositol	0.332	0.020	0.234	0.030	0.258	0.049	0.70	0.215	0.029	0.251	0.049	0.56	0.262	0.047	0.250	0.038	0.86	0.260	0.023	0.250	0.020	0.77	0.208	0.030	0.198	0.004	0.78
Nicotinic acid	0.006	0.000	0.004	0.000	0.003	0.000	0.48	0.005	0.001	0.005	0.001	0.98	0.005	0.001	0.005	0.001	0.99	0.004	0.001	0.005	0.001	0.43	0.004	0.000	0.005	0.001	0.43
Acetylserine	0.008	0.002	0.008	0.001	0.008	0.002	0.82	0.008	0.001	0.008	0.001	0.94	0.008	0.001	0.007	0.001	0.55	0.007	0.002	0.004	0.000	0.22	0.005	0.000	0.005	0.001	0.73
Octanoic acid	0.008	0.001	0.010	0.001	0.009	0.001	0.75	0.009	0.000	0.010	0.000	0.03	0.009	0.000	0.008	0.000	0.06	0.009	0.001	0.008	0.000	0.75	0.009	0.001	0.008	0.001	0.62
Ornithine	0.004	0.001	0.004	0.000	0.004	0.000	0.94	0.005	0.001	0.005	0.001	0.68	0.005	0.001	0.005	0.001	0.55	0.004	0.001	0.004	0.001	0.90	0.004	0.000	0.003	0.000	0.34
Oxalic acid	0.107	0.032	0.098	0.022	0.114	0.003	0.54	0.094	0.022	0.122	0.004	0.29	0.125	0.003	0.132	0.006	0.40	0.099	0.017	0.093	0.019	0.83	0.081	0.019	0.096	0.018	0.61
Palmitic acid	0.575	0.011	0.484	0.047	0.418	0.082	0.52	0.465	0.054	0.483	0.037	0.80	0.440	0.041	0.476	0.034	0.54	0.488	0.109	0.485	0.016	0.98	0.509	0.062	0.481	0.109	0.84
Phenylalanine	0.017	0.001	0.015	0.001	0.021	0.001	0.02	0.019	0.001	0.019	0.001	0.84	0.017	0.002	0.020	0.004	0.51	0.018	0.004	0.019	0.005	0.86	0.027	0.003	0.027	0.004	0.89
Phosphoric acid	0.018	0.002	0.014	0.001	0.013	0.002	0.82	0.016	0.000	0.016	0.002	0.83	0.024	0.010	0.024	0.008	0.99	0.014	0.002	0.013	0.002	0.70	0.010	0.001	0.010	0.002	0.99
Phytol	0.116	0.043	0.104	0.007	0.103	0.009	0.93	0.273	0.069	0.193	0.021	0.33	0.171	0.053	0.213	0.036	0.55	0.287	0.047	0.156	0.035	0.09	0.160	0.010	0.174	0.007	0.32
Pipecolic acid	0.016	0.002	0.024	0.004	0.030	0.008	0.60	0.028	0.006	0.029	0.007	0.93	0.060	0.012	0.075	0.036	0.70	0.163	0.112	0.218	0.147	0.78	0.157	0.018	0.164	0.053	0.90
Proline	1.370	0.503	1.444	0.223	1.207	0.077	0.37	2.752	0.595	2.273	0.332	0.52	3.239	0.759	2.205	0.286	0.27	3.006	0.748	4.024	0.289	0.27	9.172	1.586	7.866	2.624	0.69
Putrescine	0.045	0.008	0.068	0.005	0.065	0.002	0.64	0.055	0.003	0.056	0.002	0.83	0.061	0.006	0.072	0.018	0.60	0.095	0.028	0.109	0.028	0.74	0.196	0.048	0.220	0.056	0.76
Pyruvic acid	0.019	0.005	0.032	0.002	0.026	0.002	0.09	0.016	0.001	0.024	0.001	0.00	0.024	0.003	0.021	0.003	0.52	0.016	0.002	0.027	0.003	0.04	0.017	0.004	0.021	0.002	0.45
Raffinose	0.019	0.006	0.007	0.002	0.006	0.001	0.51	0.009	0.004	0.010	0.002	0.93	0.019	0.005	0.013	0.003	0.34	0.043	0.016	0.033	0.008	0.60	0.069	0.008	0.069	0.011	0.97
Ribose	0.037	0.002	0.033	0.001	0.034	0.003	0.69	0.034	0.006	0.037	0.004	0.63	0.037	0.002	0.039	0.002	0.49	0.038	0.007	0.037	0.002	0.90	0.050	0.003	0.045	0.008	0.60
Serine	0.377	0.207	0.644	0.025	0.748	0.063	0.20	0.863	0.052	0.892	0.130	0.85	1.043	0.146	1.184	0.215	0.62	1.130	0.259	1.258	0.234	0.73	1.040	0.124	1.013	0.126	0.89
Shikimic acid	0.073	0.007	0.067	0.002	0.069	0.006	0.80	0.069	0.003	0.077	0.004	0.19	0.065	0.003	0.075	0.007	0.24	0.053	0.008	0.061	0.007	0.54	0.037	0.002	0.037	0.005	0.98
Sinapinic acid	0.022	0.001	0.014	0.001	0.015	0.002	0.59	0.018	0.002	0.018	0.001	0.94	0.016	0.002	0.018	0.002	0.48	0.015	0.000	0.014	0.001	0.36	0.012	0.001	0.012	0.001	0.88
Spermidine	0.036	0.001	0.029	0.002	0.030	0.004	0.83	0.028	0.003	0.032	0.001	0.36	0.026	0.002	0.031	0.001	0.10	0.023	0.002	0.025	0.002	0.46	0.023	0.003	0.021	0.002	0.66
Stearic acid	0.075	0.005	0.059	0.008	0.054	0.011	0.72	0.060	0.006	0.059	0.005	0.91	0.053	0.004	0.053	0.005	0.98	0.058	0.013	0.063	0.003	0.73	0.059	0.008	0.057	0.015	0.91
Succinic acid	0.045	0.007	0.056	0.006	0.065	0.012	0.53	0.057	0.002	0.060	0.008	0.66	0.064	0.007	0.061	0.012	0.85	0.057	0.022	0.063	0.013	0.82	0.058	0.004	0.059	0.017	0.94
Sucrose	0.291	0.145	0.740	0.094	0.681	0.100	0.69	1.006	0.083	0.882	0.021	0.22	0.899	0.050	0.784	0.118	0.42	0.950	0.264	1.060	0.130	0.73	0.967	0.199	1.084	0.249	0.73
Tagatose	0.080	0.003	0.071	0.002	0.076	0.003	0.22	0.061	0.002	0.082	0.002	0.00	0.066	0.002	0.080	0.006	0.09	0.052	0.003	0.065	0.005	0.07	0.062	0.002	0.064	0.004	0.67

	0 dpi		1 dpi					2 dpi					3 dpi					5 dpi					10 dpi				
	Control		Mock LT		Bp LT			Mock LT		Bp LT			Mock LT		Bp LT			Mock LT		Bp LT			Mock LT		Bp LT		
	FC	SE	FC	SE	FC	SE	p	FC	SE	FC	SE	p	FC	SE	FC	SE	p	FC	SE	FC	SE	p	FC	SE	FC	SE	p
**Detected by GC-MS (Arbitrary unit)**																											
Tetradecanoic acid	0.019	0.002	0.016	0.002	0.016	0.003	0.95	0.016	0.001	0.019	0.001	0.06	0.014	0.001	0.018	0.001	0.12	0.015	0.002	0.017	0.002	0.45	0.017	0.002	0.018	0.002	0.69
Threonic acid	0.013	0.000	0.010	0.000	0.013	0.001	0.05	0.025	0.002	0.019	0.002	0.10	0.020	0.002	0.017	0.002	0.47	0.024	0.006	0.016	0.001	0.32	0.017	0.004	0.018	0.004	0.99
Threonic acid-1.4-lactone	0.032	0.004	0.019	0.003	0.020	0.001	0.85	0.031	0.001	0.027	0.001	0.07	0.024	0.003	0.023	0.004	0.95	0.029	0.004	0.023	0.001	0.20	0.020	0.004	0.020	0.003	0.94
Threonine	0.301	0.063	0.386	0.014	0.425	0.016	0.14	0.406	0.004	0.420	0.039	0.76	0.424	0.035	0.481	0.052	0.41	0.441	0.079	0.502	0.091	0.64	0.539	0.017	0.494	0.077	0.60
Tyramine	0.013	0.002	0.006	0.000	0.009	0.002	0.30	0.008	0.001	0.008	0.001	0.81	0.008	0.000	0.009	0.000	0.07	0.009	0.001	0.008	0.002	0.88	0.014	0.002	0.016	0.002	0.67
Tyrosine	0.008	0.001	0.004	0.000	0.007	0.001	0.15	0.006	0.001	0.006	0.001	1.00	0.006	0.000	0.007	0.001	0.29	0.009	0.003	0.009	0.002	0.93	0.013	0.001	0.021	0.008	0.41
Urea	0.004	0.000	0.003	0.000	0.004	0.000	0.05	0.013	0.008	0.004	0.000	0.40	0.004	0.001	0.004	0.000	0.74	0.003	0.001	0.004	0.001	0.86	0.005	0.000	0.003	0.001	0.08
Valine	0.291	0.030	0.267	0.015	0.318	0.052	0.39	0.308	0.021	0.300	0.018	0.78	0.323	0.017	0.345	0.033	0.58	0.338	0.050	0.353	0.038	0.83	0.572	0.084	0.547	0.087	0.85
Xylose	0.032	0.001	0.022	0.001	0.023	0.001	0.49	0.022	0.001	0.027	0.002	0.06	0.023	0.001	0.027	0.002	0.15	0.030	0.005	0.026	0.002	0.52	0.033	0.006	0.028	0.004	0.56
**Detected by LC-MS (ng per g dry weight of leaves)**																											
Caffeic acid	1411	201											1789	208	1175	125	0.06						2890	255	1620	388	0.05
Chlorogenic acid	17	12											23	9	27	4	0.75						22	5	45	18	0.29
Coumaric acid	6444	817			ND					ND			5620	790	6179	616	0.61			ND			8028	739	5104	195	0.02
Ferulic acid	1529	228											1103	104	818	105	0.13						1362	147	746	71	0.02
Hydroxybenzoic acid	488	97											354	84	500	211	0.56						1734	366	859	271	0.13

dpi, days post (bacterial) inoculation; FC, averages of fold change; ND, not determined; SE, standard error of the mean; p, p value (Student's t test).

Supplemental Table S3. Metabolite profiling in *Arabidopsis thaliana* leaves after soil drenching with PBS or *Burkholderia phytofirmans* PsJN solution

	0 dpi		1 dpi					2 dpi					3 dpi					5 dpi					10 dpi				
	Control		Mock LT		Bp LT			Mock LT		Bp LT			Mock LT		Bp LT			Mock LT		Bp LT			Mock LT		Bp LT		
	FC	SE	FC	SE	FC	SE	p	FC	SE	FC	SE	p	FC	SE	FC	SE	p	FC	SE	FC	SE	p	FC	SE	FC	SE	p
**Detected by GC-MS (relative level)**																											
Alanine	2.254	1.233	2.919	0.616	3.013	0.289	0.90	2.532	0.065	2.457	0.192	0.73	2.919	0.314	3.248	0.742	0.70	2.532	0.179	2.822	0.070	0.20	2.730	0.420	2.731	0.134	1.00
Arabinose	0.098	0.033	0.186	0.034	0.162	0.049	0.71	0.128	0.036	0.108	0.035	0.71	0.181	0.012	0.125	0.026	0.12	0.104	0.033	0.115	0.034	0.84	0.102	0.026	0.067	0.018	0.33
Ascorbic acid	1.161	0.034	1.065	0.063	1.176	0.080	0.34	0.822	0.113	1.228	0.053	0.03	0.958	0.093	1.020	0.051	0.59	1.138	0.077	1.147	0.023	0.91	0.932	0.029	0.943	0.082	0.90
Aspartic acid	0.036	0.001	0.040	0.006	0.038	0.003	0.70	0.034	0.000	0.033	0.003	0.86	0.041	0.005	0.038	0.007	0.74	0.027	0.003	0.030	0.004	0.64	0.025	0.005	0.022	0.005	0.66
Benzoic acid	0.029	0.002	0.026	0.003	0.031	0.001	0.11	0.032	0.003	0.033	0.004	0.79	0.029	0.003	0.031	0.004	0.67	0.032	0.003	0.028	0.003	0.41	0.031	0.001	0.027	0.001	0.02
Beta-Sitosterol	0.037	0.003	0.029	0.001	0.031	0.002	0.32	0.029	0.000	0.031	0.002	0.38	0.029	0.001	0.029	0.002	0.97	0.029	0.000	0.029	0.002	0.89	0.028	0.001	0.026	0.000	0.14
Citramalic acid	0.004	0.000	0.004	0.000	0.005	0.000	0.14	0.005	0.000	0.004	0.000	0.09	0.005	0.000	0.004	0.000	0.76	0.004	0.001	0.004	0.000	0.25	0.003	0.001	0.004	0.001	0.28
Citric acid	0.007	0.001	0.007	0.000	0.008	0.001	0.16	0.008	0.001	0.008	0.001	0.58	0.007	0.000	0.009	0.001	0.28	0.029	0.024	0.006	0.001	0.44	0.005	0.001	0.005	0.001	0.78
Cyclohexylamine	0.046	0.003	0.032	0.003	0.029	0.005	0.59	0.038	0.007	0.034	0.007	0.69	0.046	0.009	0.030	0.005	0.21	0.037	0.007	0.032	0.004	0.58	0.037	0.003	0.039	0.008	0.77
Allose	0.024	0.007	0.017	0.003	0.027	0.005	0.16	0.026	0.003	0.031	0.004	0.44	0.019	0.007	0.033	0.011	0.35	0.022	0.006	0.032	0.007	0.35	0.025	0.009	0.029	0.005	0.78
Digalactosylglycerol	0.067	0.011	0.028	0.001	0.035	0.010	0.58	0.037	0.004	0.045	0.006	0.32	0.043	0.004	0.045	0.004	0.76	0.050	0.008	0.057	0.002	0.40	0.080	0.011	0.090	0.011	0.59
Aminocaproic acid	0.017	0.000	0.018	0.000	0.018	0.000	0.93	0.017	0.000	0.017	0.000	0.40	0.017	0.001	0.017	0.000	0.68	0.016	0.001	0.017	0.000	0.33	0.017	0.000	0.017	0.000	0.54
Ethanolamine	1.825	0.355	0.949	0.150	1.669	0.229	0.06	1.796	0.166	2.138	0.218	0.28	0.983	0.355	1.587	0.471	0.36	1.455	0.308	1.278	0.255	0.68	1.171	0.390	1.579	0.571	0.59
Fructose	1.131	0.419	0.759	0.176	1.291	0.229	0.14	1.245	0.163	1.503	0.234	0.42	0.827	0.367	1.590	0.552	0.31	1.101	0.299	1.580	0.405	0.39	1.295	0.522	1.473	0.339	0.79
Fumaric acid	5.756	1.169	5.776	0.972	7.322	0.532	0.24	7.159	0.552	6.514	0.540	0.45	5.681	1.033	6.462	1.163	0.64	4.791	0.367	4.799	1.090	0.99	3.801	2.103	4.165	1.925	0.90
Galactinol	0.062	0.003	0.057	0.003	0.055	0.002	0.59	0.055	0.004	0.061	0.003	0.32	0.058	0.002	0.060	0.001	0.28	0.057	0.004	0.069	0.003	0.08	0.096	0.017	0.084	0.009	0.59
Galactonic acid	0.004	0.000	0.003	0.000	0.003	0.001	0.98	0.003	0.000	0.003	0.000	0.28	0.004	0.000	0.003	0.000	0.07	0.003	0.001	0.003	0.000	0.87	0.003	0.000	0.003	0.000	0.90
Galactose	0.010	0.000	0.009	0.000	0.010	0.000	0.10	0.010	0.000	0.011	0.001	0.63	0.009	0.001	0.010	0.002	0.61	0.010	0.001	0.012	0.002	0.53	0.017	0.006	0.019	0.005	0.88
Galactosylglycerol	0.070	0.017	0.039	0.002	0.044	0.013	0.72	0.057	0.009	0.071	0.012	0.40	0.042	0.001	0.053	0.010	0.38	0.048	0.010	0.054	0.008	0.71	0.072	0.013	0.089	0.013	0.39
Glucose 1	1.926	0.987	0.876	0.226	2.255	0.219	0.01	2.351	0.373	2.696	0.279	0.50	1.461	0.382	2.454	0.856	0.35	1.537	0.352	2.381	0.570	0.28	2.152	0.644	2.480	0.681	0.74
Glutamic acid	0.099	0.006	0.082	0.004	0.095	0.005	0.14	0.080	0.004	0.082	0.002	0.71	0.073	0.010	0.079	0.018	0.78	0.057	0.007	0.048	0.002	0.28	0.045	0.007	0.043	0.007	0.84
Glutamine	0.036	0.019	0.066	0.001	0.083	0.005	0.04	0.064	0.004	0.062	0.003	0.70	0.076	0.010	0.084	0.024	0.77	0.047	0.011	0.047	0.007	1.00	0.033	0.009	0.035	0.006	0.89
Glyceric acid	0.073	0.025	0.150	0.022	0.159	0.007	0.72	0.182	0.026	0.149	0.016	0.34	0.156	0.020	0.170	0.023	0.67	0.116	0.016	0.119	0.025	0.94	0.045	0.014	0.044	0.012	0.95
Glycerol	0.087	0.007	0.048	0.001	0.084	0.025	0.28	0.083	0.007	0.097	0.007	0.22	0.056	0.010	0.075	0.017	0.39	0.064	0.008	0.064	0.008	0.97	0.070	0.015	0.082	0.024	0.69
Glycine	0.046	0.025	0.135	0.029	0.170	0.016	0.35	0.148	0.016	0.159	0.054	0.85	0.135	0.032	0.177	0.091	0.69	0.081	0.018	0.108	0.028	0.47	0.057	0.021	0.057	0.013	1.00
Glycolic acid	0.005	0.000	0.009	0.001	0.007	0.001	0.16	0.009	0.001	0.008	0.001	0.49	0.010	0.001	0.006	0.001	0.08	0.006	0.001	0.009	0.001	0.10	0.005	0.001	0.006	0.002	0.60
Homoserine	0.003	0.002	0.006	0.001	0.006	0.001	0.71	0.006	0.000	0.005	0.001	0.84	0.006	0.001	0.006	0.002	0.86	0.004	0.001	0.004	0.001	0.65	0.003	0.000	0.003	0.000	0.99
IsoLeucine	0.073	0.013	0.054	0.000	0.061	0.006	0.40	0.061	0.001	0.064	0.002	0.31	0.065	0.005	0.069	0.006	0.67	0.066	0.004	0.090	0.009	0.07	0.127	0.030	0.112	0.018	0.69
Itaconic acid	0.006	0.001	0.006	0.001	0.007	0.001	0.41	0.008	0.001	0.007	0.001	0.49	0.006	0.001	0.007	0.001	0.65	0.004	0.001	0.005	0.001	0.84	0.004	0.002	0.005	0.003	0.94
Lactic acid	0.029	0.007	0.024	0.005	0.030	0.007	0.53	0.030	0.004	0.029	0.006	0.98	0.030	0.006	0.027	0.009	0.79	0.025	0.007	0.019	0.001	0.50	0.031	0.008	0.034	0.008	0.80

	0 dpi		1 dpi					2 dpi					3 dpi					5 dpi					10 dpi				
	Control		Mock LT		Bp LT			Mock LT		Bp LT			Mock LT		Bp LT			Mock LT		Bp LT			Mock LT		Bp LT		
	FC	SE	FC	SE	FC	SE	p	FC	SE	FC	SE	p	FC	SE	FC	SE	p	FC	SE	FC	SE	p	FC	SE	FC	SE	p
**Detected by GC-MS (relative level)**																											
Lauric acid	0.004	0.001	0.004	0.001	0.003	0.000	0.19	0.005	0.000	0.004	0.001	0.38	0.006	0.001	0.004	0.000	0.26	0.003	0.000	0.003	0.000	0.13	0.004	0.001	0.004	0.001	0.66
Leucine	0.047	0.011	0.024	0.002	0.030	0.003	0.15	0.028	0.001	0.033	0.000	0.06	0.032	0.004	0.035	0.006	0.72	0.036	0.001	0.057	0.009	0.14	0.094	0.027	0.084	0.019	0.79
Levoglucosan	0.061	0.028	0.092	0.015	0.080	0.026	0.71	0.071	0.023	0.054	0.026	0.65	0.099	0.010	0.069	0.018	0.23	0.061	0.021	0.063	0.023	0.96	0.054	0.019	0.028	0.015	0.35
Threitol	0.005	0.000	0.005	0.000	0.005	0.000	0.42	0.005	0.000	0.005	0.001	0.99	0.004	0.000	0.004	0.000	0.47	0.004	0.000	0.005	0.000	0.29	0.004	0.000	0.005	0.001	0.61
Lysine	0.013	0.003	0.009	0.000	0.011	0.001	0.12	0.010	0.000	0.011	0.001	0.28	0.010	0.001	0.011	0.001	0.41	0.010	0.001	0.011	0.001	0.40	0.014	0.001	0.014	0.002	0.97
Malic acid	0.048	0.003	0.049	0.002	0.048	0.004	0.89	0.055	0.008	0.051	0.004	0.68	0.061	0.006	0.057	0.009	0.76	0.043	0.002	0.053	0.004	0.09	0.045	0.015	0.053	0.013	0.72
Mannose	0.006	0.002	0.006	0.001	0.008	0.000	0.07	0.008	0.001	0.008	0.001	0.62	0.008	0.001	0.008	0.001	0.82	0.009	0.001	0.011	0.002	0.22	0.012	0.004	0.014	0.004	0.82
Melibiose	0.002	0.001	0.001	0.000	0.001	0.000	0.17	0.001	0.000	0.001	0.000	0.80	0.001	0.000	0.001	0.000	0.80	0.001	0.000	0.003	0.001	0.17	0.004	0.002	0.004	0.001	0.90
Methionine	0.005	0.001	0.009	0.001	0.010	0.001	0.58	0.010	0.001	0.009	0.001	0.63	0.010	0.001	0.009	0.002	0.95	0.006	0.001	0.007	0.001	0.30	0.005	0.001	0.004	0.001	0.56
Myo-inositol	0.332	0.020	0.268	0.021	0.305	0.040	0.46	0.298	0.020	0.291	0.038	0.88	0.283	0.024	0.306	0.047	0.69	0.283	0.044	0.269	0.017	0.78	0.268	0.019	0.267	0.024	0.98
Nicotinic acid	0.006	0.000	0.005	0.001	0.005	0.001	0.53	0.004	0.001	0.005	0.001	0.90	0.004	0.001	0.004	0.001	0.90	0.004	0.000	0.005	0.000	0.01	0.005	0.001	0.005	0.000	0.99
Acetylserine	0.008	0.002	0.007	0.001	0.007	0.001	0.98	0.008	0.001	0.008	0.001	0.94	0.008	0.002	0.007	0.001	0.55	0.005	0.000	0.005	0.001	0.78	0.002	0.001	0.004	0.000	0.14
Octanoic acid	0.008	0.001	0.009	0.001	0.011	0.002	0.48	0.011	0.000	0.009	0.001	0.11	0.011	0.001	0.011	0.001	0.82	0.008	0.001	0.009	0.000	0.05	0.008	0.000	0.010	0.000	0.01
Ornithine	0.004	0.001	0.006	0.001	0.007	0.000	0.23	0.005	0.000	0.006	0.001	0.83	0.005	0.001	0.005	0.000	0.52	0.004	0.001	0.004	0.001	0.89	0.003	0.001	0.003	0.001	0.99
Oxalic acid	0.107	0.032	0.102	0.024	0.122	0.011	0.48	0.131	0.004	0.129	0.013	0.88	0.087	0.031	0.118	0.006	0.38	0.115	0.002	0.131	0.008	0.12	0.138	0.007	0.096	0.021	0.13
Palmitic acid	0.575	0.011	0.536	0.052	0.432	0.023	0.14	0.567	0.087	0.513	0.102	0.71	0.589	0.049	0.474	0.041	0.15	0.459	0.045	0.434	0.027	0.66	0.480	0.015	0.548	0.015	0.03
Phenylalanine	0.017	0.001	0.019	0.001	0.021	0.002	0.30	0.019	0.001	0.020	0.002	0.58	0.019	0.001	0.022	0.005	0.59	0.015	0.002	0.020	0.002	0.10	0.020	0.004	0.021	0.003	0.93
Phosphoric acid	0.018	0.002	0.018	0.001	0.017	0.004	0.66	0.017	0.001	0.017	0.001	0.85	0.028	0.006	0.017	0.001	0.24	0.016	0.002	0.016	0.002	0.96	0.014	0.001	0.013	0.002	0.84
Phytol	0.116	0.043	0.147	0.018	0.141	0.020	0.83	0.120	0.037	0.113	0.035	0.89	0.150	0.039	0.170	0.066	0.81	0.109	0.040	0.127	0.037	0.75	0.113	0.027	0.086	0.017	0.45
Pipecolic acid	0.016	0.002	0.020	0.002	0.032	0.011	0.32	0.019	0.002	0.030	0.012	0.42	0.095	0.047	0.079	0.027	0.79	0.088	0.030	0.229	0.046	0.06	0.202	0.026	0.267	0.082	0.49
Proline	1.370	0.503	1.422	0.381	1.340	0.386	0.89	1.273	0.321	1.153	0.200	0.77	1.814	0.132	1.704	0.294	0.75	1.784	0.208	2.595	0.076	0.02	6.451	2.357	6.207	1.164	0.93
Putrescine	0.045	0.008	0.047	0.002	0.049	0.004	0.70	0.055	0.009	0.051	0.004	0.70	0.052	0.004	0.054	0.015	0.91	0.039	0.005	0.063	0.002	0.01	0.080	0.017	0.115	0.010	0.15
Pyruvic acid	0.019	0.005	0.023	0.001	0.026	0.002	0.26	0.024	0.003	0.022	0.002	0.47	0.027	0.004	0.027	0.004	0.95	0.024	0.001	0.027	0.002	0.32	0.019	0.002	0.018	0.002	0.64
Raffinose	0.019	0.006	0.011	0.003	0.013	0.003	0.62	0.010	0.002	0.011	0.003	0.69	0.018	0.003	0.016	0.002	0.50	0.025	0.004	0.040	0.013	0.37	0.085	0.033	0.076	0.014	0.81
Ribose	0.037	0.002	0.030	0.000	0.032	0.001	0.19	0.036	0.002	0.036	0.002	1.00	0.033	0.004	0.037	0.004	0.47	0.032	0.003	0.035	0.002	0.40	0.036	0.006	0.035	0.004	0.92
Serine	0.377	0.207	0.743	0.141	0.878	0.129	0.52	0.796	0.074	0.829	0.028	0.69	1.032	0.072	1.046	0.137	0.93	0.891	0.117	1.085	0.124	0.32	0.619	0.127	0.697	0.100	0.66
Shikimic acid	0.073	0.007	0.076	0.002	0.079	0.008	0.73	0.082	0.001	0.082	0.002	0.96	0.067	0.004	0.074	0.005	0.38	0.058	0.003	0.060	0.005	0.72	0.037	0.002	0.038	0.001	0.81
Sinapinic acid	0.022	0.001	0.023	0.001	0.025	0.002	0.31	0.022	0.002	0.021	0.002	0.73	0.021	0.001	0.020	0.000	0.24	0.017	0.003	0.018	0.003	0.92	0.014	0.001	0.012	0.001	0.10
Spermidine	0.036	0.001	0.039	0.003	0.037	0.003	0.75	0.038	0.001	0.039	0.005	0.88	0.037	0.003	0.035	0.005	0.70	0.031	0.003	0.033	0.006	0.82	0.025	0.003	0.024	0.003	0.76
Stearic acid	0.075	0.005	0.067	0.012	0.055	0.003	0.41	0.073	0.010	0.064	0.012	0.57	0.081	0.012	0.058	0.006	0.15	0.059	0.007	0.050	0.000	0.34	0.053	0.007	0.068	0.000	0.19
Succinic acid	0.045	0.007	0.051	0.006	0.054	0.003	0.75	0.063	0.003	0.060	0.004	0.59	0.057	0.003	0.071	0.018	0.49	0.048	0.006	0.056	0.007	0.45	0.057	0.004	0.065	0.008	0.40
Sucrose	0.291	0.145	0.539	0.018	0.524	0.065	0.84	0.582	0.026	0.533	0.080	0.59	0.749	0.079	0.648	0.059	0.36	0.615	0.076	0.729	0.031	0.24	0.597	0.251	0.723	0.270	0.75
Tagatose	0.080	0.003	0.072	0.004	0.081	0.007	0.35	0.059	0.008	0.083	0.004	0.05	0.064	0.006	0.073	0.003	0.29	0.078	0.005	0.082	0.001	0.46	0.064	0.002	0.065	0.006	0.88

	0 dpi		1 dpi					2 dpi					3 dpi					5 dpi					10 dpi				
	Control		Mock LT		Bp LT			Mock LT		Bp LT			Mock LT		Bp LT			Mock LT		Bp LT			Mock LT		Bp LT		
	FC	SE	FC	SE	FC	SE	p	FC	SE	FC	SE	p	FC	SE	FC	SE	p	FC	SE	FC	SE	p	FC	SE	FC	SE	p
**Detected by GC-MS (Arbitrary unit)**																											
Tetradecanoic acid	0.019	0.002	0.018	0.003	0.015	0.001	0.40	0.017	0.000	0.017	0.003	0.91	0.017	0.001	0.015	0.001	0.18	0.015	0.001	0.014	0.001	0.51	0.015	0.001	0.016	0.001	0.62
Threonic acid	0.013	0.000	0.018	0.003	0.014	0.003	0.44	0.016	0.002	0.015	0.001	0.51	0.025	0.007	0.016	0.000	0.35	0.010	0.002	0.011	0.001	0.64	0.011	0.002	0.010	0.002	0.57
Threonic acid-1.4-lactone	0.032	0.004	0.036	0.004	0.029	0.007	0.41	0.030	0.005	0.025	0.003	0.45	0.035	0.003	0.023	0.002	0.03	0.017	0.002	0.019	0.001	0.41	0.016	0.003	0.017	0.001	0.92
Threonine	0.301	0.063	0.397	0.024	0.432	0.017	0.31	0.406	0.005	0.431	0.036	0.57	0.498	0.042	0.504	0.069	0.94	0.470	0.087	0.577	0.069	0.39	0.507	0.058	0.524	0.046	0.83
Tyramine	0.013	0.002	0.008	0.001	0.009	0.001	0.55	0.009	0.000	0.009	0.001	0.74	0.009	0.000	0.009	0.001	0.65	0.009	0.001	0.011	0.001	0.29	0.012	0.001	0.012	0.002	0.97
Tyrosine	0.008	0.001	0.005	0.001	0.007	0.001	0.14	0.006	0.000	0.007	0.001	0.07	0.007	0.000	0.009	0.002	0.44	0.008	0.001	0.009	0.001	0.25	0.012	0.001	0.014	0.002	0.47
Urea	0.004	0.000	0.004	0.001	0.004	0.000	0.79	0.004	0.001	0.003	0.000	0.56	0.005	0.001	0.004	0.001	0.96	0.003	0.000	0.004	0.001	0.28	0.005	0.001	0.005	0.001	0.62
Valine	0.291	0.030	0.246	0.006	0.266	0.019	0.35	0.273	0.012	0.280	0.015	0.75	0.301	0.039	0.315	0.047	0.83	0.288	0.031	0.352	0.009	0.12	0.445	0.089	0.413	0.037	0.76
Xylose	0.032	0.001	0.029	0.001	0.032	0.003	0.44	0.029	0.000	0.030	0.001	0.12	0.029	0.002	0.030	0.002	0.76	0.025	0.003	0.026	0.002	0.85	0.021	0.002	0.022	0.003	0.87
**Detected by LC-MS (ng per g dry weight of leaves)**																											
Caffeic acid	1411	201											2581	383	2284	130	0.50						5517	956	4726	933	0.59
Chlorogenic acid	17	12											34	6	37	14	0.86						44	15	10	2	0.14
Coumaric acid	6444	817			ND					ND			8377	815	8173	778	0.87			ND			14981	1674	13738	1063	0.56
Ferulic acid	1529	228											1075	136	1025	75	0.76						1449	274	1427	330	0.96
Hydroxybenzoic acid	488	97											590	192	554	113	0.88						1483	396	1861	101	0.41

dpi, days post (bacterial) inoculation; FC, averages of fold change; ND, not determined; SE, standard error of the mean; p, p value (Student's t test).

Supplemental Table S4. Metabolite profiling in *Arabidopsis thaliana* leaves 42 days after seed immersion in PBS or *Burkholderia phytofirmans* PsJN solution

	Mock ST		Bp ST		
	FC	SE	FC	SE	p
**Detected by GC-MS (relative level)**					
Alanine	2.254	1.233	2.833	0.526	0.688
Arabinofuranose	0.098	0.033	0.143	0.047	0.483
Arabinose	0.026	0.001	0.028	0.003	0.514
Ascorbic acid	1.161	0.034	1.101	0.097	0.588
Aspartic acid	0.036	0.001	0.076	0.005	0.002
Benzoic acid	0.029	0.002	0.040	0.003	0.034
Beta-Sitosterol	0.037	0.003	0.034	0.003	0.556
Citramalic acid	0.004	0.000	0.007	0.001	0.020
Citric acid	0.007	0.001	0.015	0.003	0.060
Cyclohexylamine	0.046	0.003	0.035	0.003	0.065
Allose	0.024	0.007	0.018	0.005	0.526
Digalactosylglycerol	0.067	0.011	0.041	0.004	0.099
Aminocaproic acid	0.017	0.000	0.017	0.000	0.446
Ethanolamine	1.825	0.355	1.463	0.260	0.457
Fructose	1.131	0.419	0.768	0.257	0.501
Fumaric acid	5.756	1.169	13.260	0.454	0.004
Galactinol	0.062	0.003	0.054	0.003	0.163
Galactonic acid	0.004	0.000	0.005	0.000	0.087
Galactose	0.010	0.000	0.010	0.001	0.672
Galactosylglycerol	0.070	0.017	0.061	0.017	0.726
Glucose	1.926	0.987	1.905	0.921	0.988
Glutamic acid	0.099	0.006	0.166	0.020	0.051
Glutamine	0.036	0.019	0.253	0.048	0.014
Glyceric acid	0.073	0.025	0.213	0.003	0.028
Glycerol	0.087	0.007	0.090	0.007	0.774
Glycine	0.046	0.025	0.107	0.007	0.079
Glycolic acid	0.005	0.000	0.010	0.000	0.000
Homoserine	0.003	0.002	0.008	0.001	0.143
IsoLeucine	0.073	0.013	0.064	0.002	0.519
Itaconic acid	0.006	0.001	0.016	0.001	0.005
Lactic acid	0.029	0.007	0.024	0.002	0.528
Lauric acid	0.004	0.001	0.005	0.001	0.605
Leucine	0.047	0.011	0.029	0.002	0.182
Levoglucosan	0.061	0.028	0.057	0.024	0.914
Threitol	0.005	0.000	0.005	0.000	0.724
Lysine	0.013	0.003	0.012	0.001	0.749
Malic acid	0.048	0.003	0.112	0.003	0.000
Mannose	0.006	0.002	0.008	0.000	0.469
Melibiose	0.002	0.001	0.001	0.000	0.441
Methionine	0.005	0.001	0.018	0.002	0.003
Myo-inositol	0.332	0.020	0.308	0.016	0.406
Nicotinic acid	0.006	0.000	0.008	0.000	0.010
Acetylserine	0.008	0.002	0.007	0.001	0.928
Octanoic acid	0.008	0.001	0.011	0.001	0.036
Ornithine	0.004	0.001	0.007	0.000	0.070
Oxalic acid	0.107	0.032	0.138	0.009	0.390
Palmitic acid	0.575	0.011	0.645	0.039	0.157
Phenylalanine	0.017	0.001	0.035	0.005	0.026
Phosphoric acid	0.018	0.002	0.026	0.001	0.014
Phytol	0.116	0.043	0.191	0.064	0.388

	Mock ST		Bp ST		
	FC	SE	FC	SE	p
**Detected by GC-MS (relative level)**					
Pipecolic acid	0.016	0.002	0.012	0.001	0.155
Proline	1.370	0.503	1.053	0.082	0.568
Putrescine	0.045	0.008	0.036	0.004	0.371
Pyruvic acid	0.019	0.005	0.018	0.001	0.860
Raffinose	0.019	0.006	0.018	0.002	0.831
Ribose	0.037	0.002	0.033	0.001	0.127
Serine	0.377	0.207	0.907	0.093	0.079
Shikimic acid	0.073	0.007	0.108	0.004	0.012
Sinapinic acid	0.022	0.001	0.026	0.002	0.144
Spermidine	0.036	0.001	0.033	0.004	0.440
Stearic acid	0.075	0.005	0.086	0.008	0.279
Succinic acid	0.045	0.007	0.061	0.009	0.253
Sucrose	0.291	0.145	0.666	0.101	0.101
Tagatose	0.080	0.003	0.078	0.006	0.827
Tetradecanoic acid	0.019	0.002	0.022	0.002	0.259
Threonic acid	0.013	0.000	0.026	0.003	0.043
Threonic acid-1.4-lactone	0.032	0.004	0.038	0.003	0.275
Threonine	0.301	0.063	0.519	0.064	0.072
Tyramine	0.013	0.002	0.012	0.001	0.595
Tyrosine	0.008	0.001	0.011	0.001	0.222
Urea	0.004	0.000	0.004	0.001	0.631
Valine	0.291	0.030	0.281	0.019	0.793
Xylose	0.008	0.000	0.009	0.000	0.018
**Detected by LC-MS (ng per g dry weight of leaves)**					
Caffeic acid	1411	201	2086	245	0.100
Chlorogenic acid	17	12	31	7	0.379
Coumaric acid	6444	817	7869	673	0.250
Ferulic acid	1529	228	1331	183	0.536
Hydroxybenzoic acid	488	97	391	53	0.427

FC, averages of fold change; SE, standard error of the mean, p, p value (Student’s t test).
